# The Dual Nature of RNA Ligation and Degradation by T4 RNA Ligase 1: Insights Into an AMP‐Driven, Nucleobase‐Dependent Reverse Reaction

**DOI:** 10.1002/cbic.70559

**Published:** 2026-10-28

**Authors:** Nozomi Adachi, Wataru Takahagi, Shohei Terazawa, Ryoji F. Abe, Nao Masuda, Kosuke Fujishima

**Affiliations:** ^1^ School of Life Science and Technology Institute of Science Tokyo Meguro‐ku, Tokyo Japan; ^2^ Earth‐Life Science Institute Institute of Science Tokyo Meguro‐ku, Tokyo Japan; ^3^ Graduate School of Media and Governance Keio University Fujisawa Kanagawa Japan

**Keywords:** ligases, oligonucleotides, RNA degradation, stochastic simulation, T4 RNA ligase 1

## Abstract

T4 RNA Ligase 1 (Rnl1) catalyzes the ligation of a 5′‐phosphate to a 3′‐hydroxyl in single‐stranded RNA and is widely used in RNA biotechnology for applications such as RNA library preparation and 3′‐adapter ligation. In addition to ligation reaction, Rnl1 catalyzes the reverse reaction, leading to RNA degradation. However, how RNA sequence affects susceptibility to this reverse reaction remains unclear, and practical strategies for controlling it through buffer conditions have not been established. Previous studies showed that AMP promotes and ATP suppresses the reverse reaction; we confirmed these effects by electrophoretic analysis of FAM‐labeled RNA homopolymers, a substrate not previously examined. Furthermore, using miRNA substrates, we demonstrated that the reverse reaction can occur under conditions that more closely resemble the practical conditions in which Rnl1 is used, thereby extending the relevance of previous findings. Additionally, we showed that the Rnl1‐mediated reverse reaction exhibits pronounced nucleobase preference. The reverse reaction is suppressed by elevated temperature and duplex formation. Furthermore, experimental results combined with stochastic simulations indicate that Rnl1‐mediated degradation proceeds via an endonucleolytic mechanism. These findings define how reaction conditions and nucleobase difference influence reverse reaction efficiency and provide practical guidance for minimizing RNA degradation while maintaining ligation efficiency.

## Graphical abstract

T4 RNA Ligase 1 (Rnl1) not only ligates RNA but also catalyzes a reverse reaction leading to RNA degradation. This degradation occurs under standard ligation conditions and shows sequence preference. Here, we investigated whether the reverse reaction can be controlled by optimizing buffer conditions to improve reliability of Rnl1‐based experiments. Created in BioRender. Fujishima, K. (2026). https://BioRender.com/isi078q.

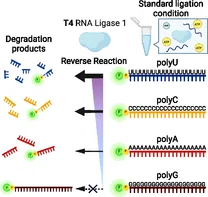

## 1 Introduction

T4 RNA Ligase 1 (Rnl1) is a widely used enzyme for ligating single‐stranded RNA [1]. It is derived from the bacteriophage T4 and catalyzes the formation of a phosphodiester bond between the phosphate group at 5′‐end and the hydroxyl group at 3′‐end of single‐stranded RNA [2]. In addition to single‐stranded RNA substrates, Rnl1 also acts on single‐stranded DNA and dinucleoside pyrophosphates, demonstrating its broad substrate tolerance [3, 4, 5]. Previous studies have further shown that Rnl1 can ligate nicked duplex substrates under appropriate conditions [6] and efficiently catalyze intramolecular circularization of short single‐stranded RNA substrates [7], demonstrating its versatility toward diverse RNA substrates in vitro. Rnl1 is used in RNA labeling [8, 9], RNA repair assays [7], adapter ligation during miRNA library preparation [10, 11], and circular RNA synthesis [2, 12, 13], underscoring its central role in RNA biology.

The forward catalytic mechanism of Rnl1 has been well‐discussed as a three‐step nucleotidyl transfer process [1, 14, 15]. Structurally, Rnl1 is composed of two functional domains: an N‐terminal nucleotidyltransferase (NTase) domain and an all helical C‐terminal domain. The NTase domain, which houses the nucleotide‐binding pocket and the active site, is highly conserved among the ligase superfamily and is sufficient to catalyze all three steps of the ligation reaction [14]. On the other hand, the C‐terminal domain of Rnl1 is not essential for the fundamental three‐step ligation mechanism (Figure 1) but is critical for specificity and enhancing the reaction rate [7]. The C‐terminal domain of Rnl1 is structurally unique and lacks homology to T4 RNA ligase 2 or other members of the ligase superfamily [16].

**FIGURE 1:**
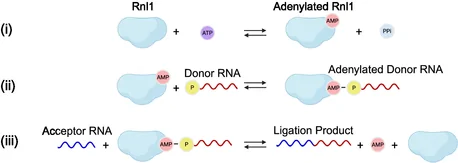
Schematic of the three‐step ligation reaction catalyzed by T4 RNA ligase 1 (Rnl1). Rnl1‐mediated ligation proceeds via three consecutive steps: (i) ATP‐dependent formation of an Rnl1‐AMP intermediate; (ii) adenylation of the phosphate at 5′‐end of the donor RNA; and (iii) RNA ligation via nucleophilic attack by the hydroxyl group at 3′‐end of the acceptor RNA with AMP release. All three steps are reversible. In the reverse reaction of the third step, RNA degradation occurs, yielding adenylated short RNA as a product. These adenylated RNAs can function as donors even in the absence of ATP. Created in BioRender, Fujishima, K. (2026). https://BioRender.com/cbemc9i.

The ligation reaction proceeds through three consecutive steps: (i) ATP‐dependent adenylation of the active‐site lysine residue of Rnl1 [15, 17] to form a covalent Rnl1‐AMP intermediate (adenylated Rnl1) with concomitant release of pyrophosphate (PPi); (ii) transfer of the adenyl group to the phosphate at 5′‐end of the donor RNA, generating an RNA‐adenylate intermediate (adenylated donor RNA); and (iii) nucleophilic attack by the hydroxyl group at 3′‐end of the acceptor RNA to form the phosphodiester bond, yielding the ligation product with release of AMP (Figure 1). Each step in this process is reversible under specific conditions [18], which underlies the versatility of Rnl1 in manipulation of RNA. Reversal of nucleotidyl transfer is not unique to Rnl1 and has also been reported for DNA ligases [19], suggesting that reverse phosphodiester bond cleavage may represent a conserved catalytic capability of ATP‐dependent nucleic acid ligases under specific conditions.

This study focuses on the reverse reaction of Rnl1, one of the least‐characterized aspects of its catalytic mechanism. The reverse reaction is stimulated by AMP and requires Mg^2+^ cation [18]. It produces adenylated RNA species that can serve as ligation‐competent donors and promote religation without ATP, effectively bypassing the initial ATP‐dependent adenylation step of Rnl1. Since Mg^2+^ and Rnl1 are routinely present in standard ligation conditions and AMP can be generated as a byproduct of ligation, reverse reactions can occur inadvertently as off‐pathway events during routine workflows. Such an unexpected degradation and religation can introduce biased sequence rearrangements and lead to incorrect data interpretation. Therefore, to reliably interpret the reaction mediated by Rnl1, it is essential to have a mechanistic and quantitative understanding of the reverse reaction, along with practical strategies for controlling it.

The aim of this study was to identify factors governing the Rnl1‐mediated reverse reaction and to establish strategies for its suppression. We first quantified nucleobase‐dependent differences in reverse reaction efficiency using 20 nt RNA homopolymers (polyU, polyC, polyA, and polyG), demonstrating that the reaction occurs under standard ligation conditions with pronounced nucleobase preference. We next examined the effects of ATP and AMP concentrations, incubation time, temperature, and duplex formation, showing that reverse reaction efficiency is strongly condition‐dependent and can be modulated. Comparison of the results of the time‐course experiment with stochastic kinetic modeling based on the Gillespie algorithm enabled prediction of time‐resolved degradation product distributions and allowed discrimination between endonucleolytic and exonucleolytic mechanisms. Collectively, these findings establish conditions that suppress the reverse reaction, propose degradation mechanisms, and offer a foundation for controlling Rnl1‐mediated chemistry in ligation‐based applications.

## 2 Results and Discussion

### 2.1 RNA Homopolymer Degradation Under Standard Ligation Conditions

To examine the reverse reaction under standard ligation buffer conditions (1 mM ATP, 50 mM Tris‐HCl (pH 7.5), 10 mM MgCl_2_, and 10 mM DTT), we performed assays using RNA homopolymers. To exclude operator‐derived RNase contamination, reactions were also performed in the presence of an RNase inhibitor. To monitor RNA degradation, the 5′‐end of each 20 nt homopolymer was labeled with FAM (6‐carboxyfluorescein) and reaction products were visualized by direct fluorescence imaging. This approach provided higher sensitivity and sharper band resolution than conventional nucleic‐acid staining, enabling detection of 1 nt differences in fragment length. FAM‐labeled homopolymers (FAM‐U20, FAM‐C20, FAM‐A20, and FAM‐G20) were mixed with the corresponding 5′‐phosphorylated homopolymers (pU20, pC20, pA20, and pG20) and incubated with or without Rnl1 and RNase inhibitor (Figure 2). In this setup, the FAM‐labeled homopolymers and 5′‐phosphorylated homopolymers act as acceptors and donors, respectively. All homopolymers used in this study are listed in Table S1.

**FIGURE 2:**
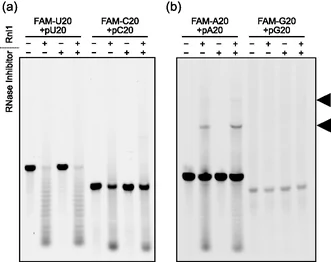
Observation of the forward/reverse ligation of RNA homopolymers. (a) Reaction of 20 nt polyU and polyC substrates by Rnl1 under 1 mM ATP conditions. (b) Reaction of 20 nt polyA and polyG substrates by Rnl1 under 1 mM ATP conditions. All reactions were performed at 16 °C for 1 h. In each sample, the most prominent band corresponds to the 20 nt RNA. Bands above 20 nt represent ligation products, whereas bands below correspond to degradation products. Observable ligation products of polyA are indicated by black arrows. Degradation products can be observed as ladder bands in single‐nucleotide resolutions below the 20 nt bands.

Because electrophoretic mobility in polyacrylamide gels can be influenced by base composition, homopolymers of identical length do not necessarily comigrate on denaturing PAGE. This effect has been demonstrated for oligonucleotides, and related base‐composition‐dependent mobility shifts have also been reported for RNA under certain electrophoretic conditions [20, 21]. In our experiments, each homopolymer showed different electrophoretic mobility, and the most prominent bands corresponded to the 20 nt substrates.

Under the conditions tested, bands lower than 20 nt consistent with degradation products appeared for polyU, polyC, and polyA. Control reactions lacking Rnl1 (Figure 2; Rnl1−) showed no degradation, whereas it was still observed in the presence of RNase inhibitor (Figure 2; RNase Inhibitor+). Furthermore, the distribution of these degradation products differed from that generated by RNase A (Figure S1), indicating that the observed degradation pattern was distinct from that caused by RNase A cleavage. This indicates that the observed degradation is Rnl1‐dependent reverse reaction rather than caused by contamination from operator‐derived RNases. Although ligation products were not detected for polyU and polyC under the conditions shown in Figure 2, intermolecular ligation products were observed for both polyU and polyC when a donor carrying a 3′‐terminal ddC modification was used (Figure S2). Because the 3′‐terminal ddC modification prevents circularization, this experiment allowed us to evaluate intermolecular ligation separately from intramolecular ligation. These results suggest that circularization occurred under the conditions shown in Figure 2, which may have prevented the detection of intermolecular ligation products. Under 1 mM ATP conditions, the decrease in substrate followed the order polyU > polyC > polyA (Figure 2). Since substrate reduction can result from either ligation or degradation, the decrease in substrate for polyU and polyC, for which no ligation products were detected, is considered to primarily reflect degradation efficiency. Under donor‐absent 20 μM AMP conditions, where ligation cannot occur, a similar trend was observed, although the difference in degradation efficiency between polyU and polyC was less pronounced than under ATP conditions (Figure S3). The reason for this difference remains unclear. However, under ATP conditions, the observed substrate reduction may be influenced by the efficiency of the initial ligation‐related process, including donor circularization, which may affect the generation of AMP required for progression of the reverse reaction. In contrast, under AMP conditions, where AMP is supplied at the same concentration from the beginning of the reaction, the observed differences may more directly reflect sequence‐dependent differences in degradation efficiency arising from substrate properties, such as structural accessibility and stability.

Comparison of the degradation products with a FAM‐U5 marker indicated that the shortest detectable products migrated at approximately the same position, corresponding to a length of 4–5 nt (Figure S4). Previous studies have established that the minimum substrate length required for ligation differs between the donor and acceptor molecules; specifically, the minimal donor is a nucleoside‐3′,5′‐diphosphate, whereas the minimal acceptor requires a trinucleotide [1]. In the absence of a 3′‐phosphate, a dinucleotide possessing a 5′‐phosphate can serve as the minimal viable donor. Consequently, combining this 2 nt donor with the minimal 3 nt acceptor yields the shortest possible religation product of 5 nt. Based on these observations, the apparent lower limit of around 4–5 nt observed for our degradation products may be consistent with the structural constraints of this reverse ligation reaction. However, alternative explanations cannot be excluded, and further investigation is necessary to fully substantiate this mechanism.

In addition, bands likely corresponding to 40 and 60 nt (▶ in Figure 2b) were observed for polyA, which are likely to represent ligation products. Also, faint smeared bands migrating above the 20 nt substrate were detected for all homopolymers (Figure 2). These smeared bands likely represent religation products formed from adenylated degradation fragments.

PolyG exhibited resistance to both degradation and ligation. This behavior is plausibly explained by formation of stable structure called G‐quadruplex (G4) [22, 23], which can limit enzyme accessibility. RNA‐degrading enzyme, such as RNase T1, exhibit reduced activity when G4 structures form, likely due to limited substrate accessibility [24]. Consistent with this hypothesis, degradation was observed when a DNA‐RNA chimera, which has a reduced propensity to form G4 structures, was used as the substrate (Figure S5). Guanine is also known to quench FAM fluorescence [25], likely reducing signal intensity and making electrophoretic visualization more difficult for polyG sequences than for other homopolymers. To address this, SYBR Green II staining was evaluated. However, Qubit‐based analysis revealed that SYBR Green II staining exhibits a strong sequence bias among the homopolymers (Figure S6). Furthermore, even with SYBR Green II staining, no detectable ligation or degradation was observed for polyG (Figure S7).

### 2.2 Effect of Excess ATP Conditions

Previous studies mentioned that high ATP concentrations suppress the reverse reaction [1, 18]. This is likely because excess ATP promotes the adenylation of Rnl1 (Figure 1i), resulting in reduced availability of free Rnl1 required for the reverse reaction. While previous studies have focused on the transfer reaction of short RNAs [18], longer and simpler RNAs were examined in this study to better assess changes in degradation efficiency under conditions more closely reflect practical use of Rnl1. We examined ATP concentrations of 1, 5, and 10 mM to determine whether ligation occurs under conditions in which degradation is suppressed. Reactions were performed in both donor‐present (Figure 3) and donor‐absent (Figure S8) conditions. Since the reverse reaction does not proceed in the absence of magnesium (Figure S9), ATP was supplied as a magnesium salt to prevent magnesium chelation.

**FIGURE 3:**
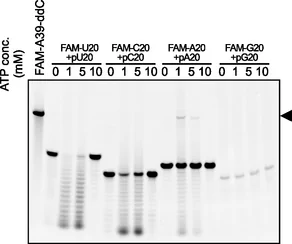
Assays evaluating the effect of ATP concentrations of 0, 1, 5, and 10 mM in the presence of a donor substrate harboring the same nucleobase as the acceptor. At 10 mM ATP, degradation was reduced compared with the levels observed at 1 and 5 mM ATP. However, the ligation product detected in the polyA sample at 1 mM ATP was not observed at 10 mM ATP. The ligation products of polyA are indicated by black arrows.

As a result, for polyU and polyC substrates, degradation efficiency was highest at 1 mM ATP and decreased with increasing ATP concentration (Figure 3). This result is consistent with previous studies showing that high ATP concentrations suppress the reverse reaction [1, 18]. Also, clear ligation products were not observed even under the conditions that suppressed the reverse reaction. For polyA, ligation products were detected at 1 mM ATP, but the ligation efficiency decreased at 5 mM and was abolished at 10 mM. This suppression of ligation under conditions of excess ATP is consistent with previous studies [26]. For polyG, neither degradation nor ligation was observed, showing the same trend as in Figure 2. To further examine the ATP dependance of the reverse reaction, reactions were also performed at lower ATP concentrations (0, 2, 20, and 200 μM) (Figure S10). No degradation was observed in the absence of ATP, whereas degradation was detected in the presence of ATP, confirming that ATP is required for the reverse reaction. Under donor‐absent conditions, minimal degradation signals were detected, although no ligation could occur, and such degradation is not expected (Figure S8).

These results suggest that ATP concentrations sufficient to completely suppress the degradation also prevent ligation. Taken together, these findings indicate that a tradeoff exists between suppression of the reverse reaction and maintenance of ligation efficiency, highlighting the importance of optimizing ATP concentration and exploring additional conditions to further suppress the reverse reaction.

### 2.3 Effect of Excess AMP Conditions

Previous studies have reported that AMP promotes the reverse reaction [18]. However, how the efficiency of the reverse reaction changes with AMP concentration has not been clearly characterized. Therefore, we examined reverse reaction efficiency under increasing AMP concentrations (0, 2, 20, and 200 μM) using FAM‐U20, FAM‐C20, FAM‐A20, and FAM‐G20 (Figure 4).

**FIGURE 4:**
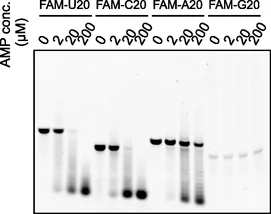
Effect of AMP concentration on the Rnl1‐mediated reverse reaction. Reactions were performed with 0, 2, 20, and 200 μM AMP. Reverse reaction efficiency increased with increasing AMP concentration for FAM‐U20, FAM‐C20, and FAM‐A20. In contrast, FAM‐G20 showed no detectable degradation even at the 200 μM AMP concentration tested.

As a result, degradation of FAM‐U20, FAM‐C20, and FAM‐A20 became progressively more apparent as the AMP concentration increased. When excess AMP was added (e.g., 200 μM), extensive degradation was observed for polyU and polyC to the extent that the 20 nt starting material band was no longer detectable. This result indicates that the reverse reaction can proceed very efficiently under certain conditions. Furthermore, efficient degradation was also observed for polyA, which had not been detected under ATP‐containing conditions (Figure 3). These results are consistent with a previous study [18] reporting that AMP promotes the reverse reaction.

In addition, small amounts of religation products (smear bands) were observed above the 20 nt substrate in several samples of polyU, polyC, and polyA (e.g., polyA with 20 μM AMP). These smear bands were also observed under 1 mM ATP conditions (Figure 2) and ATP‐excess conditions (Figure 3) but were detected here even in the absence of ATP. This observation supports a previous study [18] reporting that degradation products are adenylated and that adenylated RNA can undergo religation in the absence of ATP.

In contrast, polyG still showed no detectable degradation even under conditions in which excess AMP was added. This suggests that the resistance to degradation is not an intrinsic property of the guanine itself but is more likely due to the formation of G4 structures, as suggested in Figure 2.

### 2.4 Temperature Effects on Reverse Reaction Efficiency

To evaluate the effect of temperature on the reverse reaction, incubations were performed at 16, 23, 30, and 37 °C. All reactions were conducted in the presence of 200 μM AMP, a condition that supports efficient degradation. As a result, the efficiency of the reverse reaction was significantly reduced at 37 °C (Figure 5). This decrease is unlikely to be due to enzyme inactivation, as previous studies have demonstrated that Rnl1 retains ligation activity at 37 °C [27]. In addition, for the FAM‐A20 substrate, a stepwise increase of the reverse reaction was observed between 16 and 30 °C.

**FIGURE 5:**
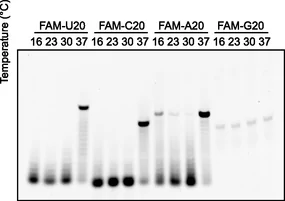
Effect of temperature on the Rnl1‐mediated reverse reaction. Reactions were performed at 16, 23, 30, and 37 °C in the presence of 200 μM AMP. Reverse reaction efficiency increased up to 30 °C and was reduced at 37 °C for polyU, polyC, and polyA. PolyG showed no detectable degradation under any temperature conditions.

These results indicate that the forward and reverse reactions do not necessarily share the same optimal temperature and suggest that the reverse reaction is selectively suppressed under high‐temperature conditions. Although the mechanism underlying this suppression remains unclear, one plausible interpretation is that increasing temperature affects the conformational dynamics of the enzyme and/or the efficiency of substrate binding.

Another possible explanation is related to changes in buffer conditions. Tris‐HCl is included in the general composition of currently used ligation reaction buffers and exhibits temperature‐dependent changes in pH [28, 29]. Such changes have been reported to affect the ligation efficiency of Rnl1 [6]. Although the behavior of the reverse reaction under different pH conditions remains unclear, we therefore repeated the experiments using MOPS buffer, which exhibits minimal temperature‐dependent pH changes (Figure S11). Similar overall trends were observed, suggesting that the suppression of degradation observed at 37 °C cannot be explained solely by temperature‐dependent changes in the pH of the reaction solution.

The forward ligation reaction requires alignment of the RNA 3′‐end with the activated 5′‐adenylated end, positioning the hydroxyl group of 3′‐end for nucleophilic attack. In contrast, the reverse reaction and deadenylation require proper positioning of both the adenylate and an internal phosphodiester bond of the RNA within the active site. Structural studies indicate that Rnl1 contains an NTase domain with active site lysine forming the covalent ligase‐AMP intermediate and a C‐terminal domain involved in substrate recognition [27, 30]. These domains provide the framework that enforces the necessary alignments. Increased thermal motion at elevated temperature may reduce the lifetime of the Rnl1‐RNA complexes required for reversal, thereby shifting the kinetic partitioning toward forward reaction. Further experiments that separately probe individual reaction steps (e.g., using preadenylated substrates) would help localize the temperature‐sensitive transitions.

### 2.5 Influence of Complementary Strand

The influence of substrate secondary structure on how the reaction changed over time under 1 mM ATP conditions was examined. We focused on polyU as ligation has not been observed for this substrate and it showed the most pronounced degradation. In this experiment, the ligation of FAM‐U20 and pU20 was examined by adding either A20 (unmodified 20 nt polyA) or U20 (unmodified 20 nt polyU) to test how duplex formation affects the reaction.

When FAM‐U20, pU20, and A20 were mixed, the duplex formation markedly suppressed the reverse reaction. Under these conditions, the expected 40 nt ligation product accumulated over time (▶ in Figure 6). In contrast, when FAM‐U20, pU20, and U20 were mixed, progressive degradation was observed. In samples undergoing reverse reaction, smear‐like bands above 20 nt were observed, consistent with religation events involving adenylated RNA fragments derived from degradation.

**FIGURE 6:**
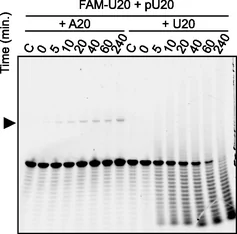
Effect of complementary strand formation on the Rnl1‐mediated reactions. Lane C represents negative control in which EDTA was added prior to Rnl1 addition. Reactions were performed at 16 °C, and products were analyzed at 0, 5, 10, 20, 40, 60, and 240 min. The ligation products of polyU are indicated by black arrow. In the presence of the complementary strand (+A20), degradation was suppressed and the expected ligation products accumulated over time. In contrast, in the absence of the complementary strand (+U20), progressive degradation was observed, and smear‐like bands above 20 nt were detected, consistent with religation of adenylated degradation fragments.

These observations indicate that duplex formation reduces susceptibility to Rnl1‐mediated degradation and promotes ligation, potentially via splinting with the complementary strand. Mechanistically, base pairing likely constrains terminal flexibility and limits access of the ligase active site to internal phosphodiester bonds. This enhanced stability creates plausible conditions that are favorable for forward ligation.

In addition, when A20 is present, the A20 strand itself can potentially function as an acceptor. Although the A20 strand cannot be directly detected in the present assay because it lacks a fluorescent label, the possibility that it functions as an acceptor strand should be considered when interpreting these results. To prevent such ligation involving A20, it may be effective to modify the hydroxyl group at 3′‐end (e.g., replacing it with amino group) or to introduce 2′,3′‐dideoxy modifications so that the strand can no longer function as an acceptor.

### 2.6 Implications of Stochastic Modeling for the Reverse‐Reaction Mechanism

To predict the mechanism of Rnl1‐mediated RNA degradation, time‐dependent cleavage profiles were analyzed using FAM‐U20 (Figures 7a and S12). Gel electrophoresis revealed progressive depletion of the 20 nt substrate accompanied by accumulation of shorter fragments. Quantitative analysis of defined fragment lengths (5, 10, 15, 20, and 22 nt species) (Figure S12) provided a time‐resolved profile of polymer length redistribution (Figure 7a). To interpret these distributions mechanistically, we considered three alternative kinetic scenarios (Figure 7b): a nonprocessive exo‐mode involving sequential 3′‐to‐5′ nucleotide removal [31, 32], and a processive exo‐mode characterized by rapid multi‐nucleotide cleavage following 3′‐end recognition [33], and an endo‐mode involving internal phosphodiester bond cleavage [34]. Each catalytic model predicts a distinct temporal pattern of fragment accumulation.

**FIGURE 7:**
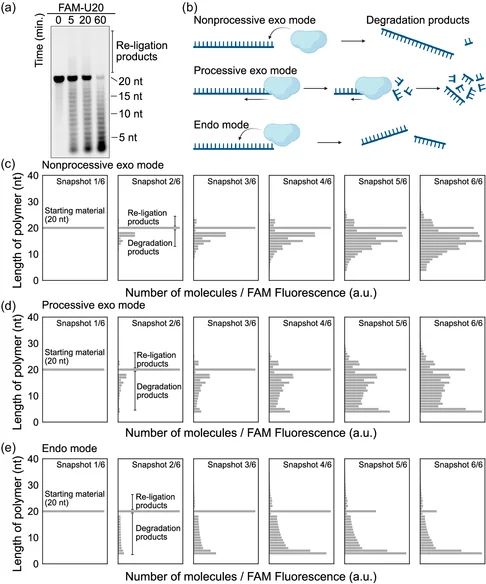
(a) Assays evaluating the Rnl1‐mediated degradation of FAM‐U20 at 0, 5, 20, and 60 min, performed at 16 °C in the presence of 20 μM AMP. (b) Schematic overview of three proposed reverse‐reaction pathways. Created in BioRender. Fujishima, K. (2026). https://BioRender.com/3ats2va. (c–e) Stochastic simulations of the coupled degradation and ligation processes (see the Figure S12 regarding the calculation method). Panels c, d, and e correspond to the nonprocessive exo‐mode, processive exo‐mode, and endo‐mode, respectively. In the simulations, degradation and ligation were implemented as stochastic reaction events, and the system state was sampled at intervals of 0.01 over a total simulation time of 75. For each mode, 1000 independent trajectories were generated, and the bars shown represent the mean values across these runs. The resulting time‐courses were divided into six segments, and representative snapshots are shown to visualize the evolution of product distributions over the simulation trajectory.

Stochastic simulations based on the Gillespie algorithm [35, 36] were performed for each model, and the resulting time‐course distributions were compared with experimental data (Figure 7c‍–‍e). The state of the system was recorded at intervals of 0.01 over a total simulation time of 75. For each mode, 1000 independent trajectories were simulated, and the values shown as bars correspond to the mean across these runs, yielding robust estimates that capture stochastic variability.

In the nonprocessive exo‐mode simulation (Figure 7c), once the 20 nt starting material began to degrade, degradation products shorter than 20 nt started to appear. The median of the length distribution did not change abruptly; instead, the overall distribution largely retained its shape while gradually shifting toward shorter lengths. In contrast, in the processive exo‐mode simulation (Figure 7d), short degradation products predominated compared with the nonprocessive exo‐mode because a single Rnl1‐mediated degradation event generated shorter fragments. In the endo‐mode simulation (Figure 7e), the distribution of degradation products was more regular than in the two exo‐mode simulations, showing a clear trend in which shorter fragments were more abundant. The distribution of religation products was also readily explained by the fact that Rnl1‐mediated cleavage is accompanied by adenylation, and the resulting adenylated fragments can serve as ATP‐independent donors. Focusing on the 22 nt species as a representative religation product, the simulation predicted that it began to appear after degradation of the 20 nt starting material and then decreased as degradation progressively dominated (Figure S12). This transient behavior was a shared feature of the processive exo‐mode and the endo‐mode. In comparison to our experimental data, the endo model most closely recapitulated the observed fragment profiles (Figures 7a and S12d), reproducing the concurrent accumulation of multiple fragment lengths and the relative enrichment of longer intermediates. Especially, the endo‐mode simulation reproduces the time‐dependent changes in both degradation and religation well. In particular, snapshots 2/6, 4/6, and 6/6 of the endo‐mode simulation appear to closely recapitulate the length distributions obtained from Rnl1‐mediated degradation of FAM‐U20 at 5, 20, and 60 min, respectively.

Taken together, these results support a model in which Rnl1‐mediated RNA degradation proceeds via an endonucleolytic pathway. This behavior is mechanistically consistent with microscopic reversal of the third ligation step (Figure 1iii), whereby AMP‐dependent chemistry acts on preformed phosphodiester bonds. Under the tested conditions, the reverse reaction thus manifests as an intrinsic AMP‐driven endonuclease activity of Rnl1.

## 3 Conclusion

In this study, we systematically investigated the reverse reaction catalyzed by Rnl1 and demonstrated that its efficiency is strongly dependent on RNA sequence and reaction conditions. While previous study established the existence of Rnl1‐mediated RNA degradation as the reverse of the ligation step [18], our results provide a more comprehensive view by revealing pronounced nucleobase‐dependent differences and identifying practical conditions that suppress this reaction. Notably, by analyzing product distributions, performing computational analyses, and conducting experiments under conditions that more closely reflect practical Rnl1 applications, this work contributes to a broader understanding of the Rnl1‐catalyzed reverse reaction. Through the analyses conducted in this study, we demonstrated that RNA degradation mediated by the reverse reaction of Rnl1 occurs even under conventional ligation conditions. Furthermore, the reverse reaction was suppressed under ATP‐excess conditions (Figure 3) but enhanced under AMP‍‐‍excess conditions (Figure 4). The results obtained under AMP‍‐‍excess conditions further revealed that RNA degradation by the reverse reaction exhibits sequence preference. Sequence‐dependent differences were observed in the Rnl1 reverse reaction among RNA substrates (Figure 2). Quantitative analysis of substrate reduction further supported the existence of sequence‐dependent differences in degradation efficiency (Figure S3). In addition, analysis of reaction temperature showed that the reverse reaction was promoted up to 30 °C but was suppressed at 37 °C (Figure 5). Furthermore, under conditions including a complementary strand, the reverse reaction was suppressed, and ligation was observed even between uridine residues (Figure 6). Finally, stochastic simulations suggested that Rnl1‐mediated cleavage proceeds predominantly via an endo‐mode mechanism (Figure 7). This endo‐mode behavior provides a consistent explanation for the experimentally observed time‐dependent evolution of product distributions, including the gradual accumulation of religation products. Furthermore, the reverse reaction was also observed when using four different miRNAs as substrates (Figure S13). We found clear differences in cleavage rate and pattern indicating that reverse reaction could occur against natural RNA sequences under standard ligation conditions therefore not limited to specific de novo sequences. While our tested RNA samples are still limited, particular attention should be paid when processing RNA with Rn1 due to its nature of sequence‐dependent cleavage, especially for regions containing consecutive U or C residues, as shown in the case of miR‐145. Collectively, the findings of this study are expected to improve the reliability of experiments using Rnl1 by promoting ligation while minimizing unintended RNA degradation.

## 4 Experimental Section

### 4.1 Materials

For all experiments in this study, unless otherwise noted, the following reagents were used: RNA sequences (FASMAC), 50% (w/v) PEG 8000 (NEB), Rnl1 (TAKARA), 10× T4 RNA Ligase buffer (500 mM Tris‐HCl pH 7.5, 100 mM MgCl_2_, 100 mM dithiothreitol, and 10 mM ATP; TAKARA), and HyClone HyPure Water (Cytiva). The PEG concentration assay is shown in Figure S14.

### 4.2 Standard Ligation Condition

Reactions were prepared to a final volume of 10 μL, containing 25% PEG 8000 and 40 U of Rnl1. The 10× T4 RNA Ligase buffer was diluted to a final 1× concentration. To quench the reaction, 0.4 M sodium ethylenediaminetetraacetate (EDTA, pH 8.0; FUJIFILM Wako) was added at 1 μL per reaction, as Mg^2+^ is required for the reverse reaction (Figure S9). The final concentration of each RNA polymer was adjusted to 3.3 μM. Reactions were carried out in a thermal cycler (T100 Thermal Cycler; Bio‐Rad) at 16 °C for 1 h. The reaction conditions were established based on the manufacturer’s recommended protocol and were further optimized empirically. The reaction products were analyzed by electrophoresis.

### 4.3 Electrophoresis

After incubation, the products were visualized by electrophoresis. For each sample, 1 μL (approximately 22 ng, 3.3 pmol) was used. Samples were mixed with 1 μL of 2× RNA loading dye (95% Formamide, 0.02% sodium dodecyl sulfate, 0.02% bromophenol blue, 0.01% Xylene Cyanol, and 1 mM EDTA, NEB) and denatured into single‐stranded RNA by heating at 65 °C for 30 s in the thermal cycler. Electrophoresis was performed on a SuperSep RNA 15%, 17 wells (FUJIFILM Wako) using 1× TBE buffer at 200 V for 60 min or 10 mA for 55 min. Fluorescence images of the gel were captured using an Amersham Imager 680 (GE Healthcare) using fluorescent blue (480 nm) mode.

### 4.4 Evaluation of the Reverse Reaction of Rnl1

Twenty‐nucleotide RNA homopolymers (polyU, polyC, polyA, and polyG) with 6‐FAM labeling at 5′‐end (FAM‐U20, FAM‐C20, FAM‐A20, and FAM‐G20) were each mixed with their corresponding 5′‐phosphorylated donors (pU20, pC20, pA20, and pG20) in a total reaction volume of 20 μL. Reactions included 1 μL of RNase Inhibitor (SUPERase⋅In RNase Inhibitor, 20 U/μL, Invitrogen) and were incubated under the conditions described in the Standard ligation conditions section. Reactions were stopped by adding 2 μL of EDTA.

### 4.5 Excess ATP Conditions

The effect of excess ATP on the reverse reaction was investigated using FAM‐U20, FAM‐C20, FAM‐A20, and FAM‐G20 with corresponding donor RNA. In this study, ATP (magnesium salt, Sigma–Aldrich) concentrations of 1, 5, and 10 mM were tested to determine how the extent of the reverse reaction varies with ATP concentration. ATP concentrations of 5 and 10 mM were prepared by adding 4 or 9 mM ATP to T4 RNA Ligase buffer (TAKARA), which contains 1 mM ATP.

### 4.6 Excess AMP Conditions

For each of FAM‐U20, FAM‐C20, FAM‐A20, and FAM‐G20, the reverse reaction was examined under excess AMP conditions (0, 2, 20, and 200 μM). To specifically evaluate the reverse reaction, phosphorylated donor RNAs and ATP were not included in this experiment. Reactions were prepared using 10× basal buffer without ATP (500 mM Tris‐HCl pH 7.5, 100 mM MgCl_2_, and 100 mM dithiothreitol). The composition of the 10× basal buffer was based on the 10× T4 RNA Ligase buffer (TAKARA). An equal volume of AMP solution (Sigma–Aldrich) was mixed with the buffer to prepare a 5× AMP buffer, which was then diluted to a 1× working concentration in the reaction mixture.

### 4.7 Investigation of Temperature Dependance

We investigated the effect of reaction temperature on the efficiency of the reverse reaction of FAM‐U20, FAM‐C20, FAM‐A20, and FAM‐G20. Samples were incubated at 16, 23, 30, or 37 °C. The reaction was carried out using 5× AMP buffer diluted to 1× working concentration in the reaction, giving a final AMP concentration of 200 μM.

### 4.8 Complementary Strand Experiments

To examine how the formation of complementary strands influences both forward and reverse reactions, we prepared two mixtures: one containing FAM‐U20, pU20, and A20 (which can form complementary duplexes) and another in which U20 was used instead of A20 (which cannot form complementary strands). For each sample, we assessed the effect of incubation time by measuring reactions from 0 min (negative control) to 240 min.

### 4.9 Stochastic Simulation

To quantitatively evaluate the candidate degradation mechanisms of Rnl1, we performed stochastic simulations using the Gillespie algorithm. The simulations explicitly tracked the molecular counts of FAM‐labeled and adenylated RNA species, incorporating the ligation model and three distinct degradation models: nonprocessive exo‐mode, processive exo‐mode, and endo‐mode. All models and simulations were implemented using an in‐house Python script. For further details, see S12 and Table S2.

## Author Contributions

**Nozomi Adachi**: data curation (lead), methodology (lead), investigation (lead), visualization (lead), and writing – original draft (lead). **Wataru Takahagi**: conceptualization (lead), project administration (equal), methodology (equal), investigation (lead), visualization (equal), writing – original draft (equal), and supervision (equal). **Shohei Terazawa**: conceptualization (supporting), methodology (equal), investigation (lead), visualization (equal), and writing – review, and editing (equal). **Ryoji F. Abe**: methodology (equal), investigation (equal), visualization (supporting), and writing – review, and editing (supporting). **Nao Masuda**: investigation (supporting), writing – review, and editing (equal). **Kosuke Fujishima**: project administration (equal), funding acquisition (lead), methodology (supporting), investigation (supporting), resources (lead), writing – review, and editing (equal), and supervision (lead).

## Funding

This study was supported by the Toyota Konpon Research Institute (JY255001Mn).

## Conflicts of Interest

The authors declare no conflicts of interest.

## Supporting information

Supplementary Material

## Data Availability

All data that supporting the findings of this study are available in the article and Supporting Information files. Source codes for stochastic simulations based on the Gillespie algorithm in Section 2.6 are available at Zenodo at https://doi.org/10.5281/zenodo.19162391. BioRender was used for graphical abstract, Figure 1 and Figure 7.
