# Supplementary material for: The Dual Nature of RNA Ligation and Degradation by T4 RNA Ligase 1: Insights Into an AMP‐Driven, Nucleobase‐Dependent Reverse Reaction
Source: Chembiochem. 2026 Oct 28;27(21):e70559. doi: 10.1002/cbic.70559 (PMC13717052; doi:10.1002/cbic.70559)
Supplement: Supplementary file 1 [file CBIC-27-e70559-s001.pdf]

Supplementary Information for

The Dual Nature of RNA Ligation and Degradation by T4 RNA ligase 1: Insights into an AMP-driven, Nucleobase-dependent Reverse Reaction

Nozomi Adachi,[a] Wataru Takahagi,*[b] Shohei Terazawa,[a] Ryoji F. Abe,[a] Nao Masuda,[b] Kosuke Fujishima*[b,c]

[a] School of Life Science and Technology, Institute of Science Tokyo, 2-12-1 Ookayama, Meguro-ku, Tokyo 152-8550, Japan.

[b] Earth-Life Science Institute, Institute of Science Tokyo, 2-12-1 Ookayama, Meguro-ku, Tokyo 152-8550, Japan.

[c] Graduate School of Media and Governance, Keio University, 5322 Endo, Fujisawa, Kanagawa 252-0816, Japan.

Contents

1 Comparison of degradation product distributions generated by RNase A and the Rnl1 reverse reaction 3

2 Evaluation of ligation and degradation efficiencies using ddC-modified RNA donors 3

3 Quantification of the residual 20 nt polyU, polyC, and polyA substrates 3

4 Estimation of the minimum degradation product length using 5 nt FAM-labeled polyU 3

5 Evaluation of G residue degradation using DNA-RNA chimeric substrates 4

6 Quantification of the staining efficiency of SYBR Green II for RNA homopolymers using a Qubit assay 4

7 Post-staining to detect products lacking the FAM label 4

8 Unexpected RNA degradation in the absence of donor RNA under excess ATP conditions 4

9 Verification of the reverse reaction under different buffer conditions with or without ATP, AMP, and Mg^{2+} 5

10 Effects of low ATP concentrations on ligation and degradation by Rnl1 5

11 Temperature dependence of the Rnl1 reverse reaction under pH-stable conditions using MOPS buffer 5

12 Stochastic simulation based on the Gillespie algorithm 5

12.1 Simulation model for RNA ligation and degradation . . . 5

12.2 Stochastic simulation using Gillespie algorithm . . . 6

12.3 Simulation setup and computational parameters . . . 7

13 Verification of the Rnl1 reverse reaction using miRNA substrates 7

14 Evaluation of the reverse reaction efficiency as a function of PEG concentration 7

List of Figures

S1 Comparison of the distribution of reaction products generated by RNase A and Rnl1 . . 8
S2 Analysis of the reverse reaction under circularization-deficient conditions using a ddC-modified donor 9
S3 Analysis of substrate reduction under donor-absent 20 µM AMP conditions 10
S4 Comparison of the migration of FAM-U5 and Rnl1 reaction products 10
S5 Degradation of guanine-containing chimeric DNA-RNA substrates 11
S6 Evaluation of the sequence-dependent fluorescence bias of SYBR Green II using a Qubit fluorometer 11
S7 SYBR Green II post-staining of the gel shown in Figure 2 12
S8 Control assays evaluating the effects of ATP concentration in the absence of a donor substrate 12
S9 Effects of ATP, AMP, and Mg on the reverse reaction 13
S10 Effects of low ATP concentrations on ligation and degradation using a donor with a 3′-terminal ddC modification 14
S11 Temperature dependence of the Rnl1 reverse reaction in MOPS buffer 14
S12 Stochastic simulations of Rnl1-mediated reverse-reaction modes and quantitative gel-based kinetics 15
S13 Ligation and degradation of miRNAs under AMP and ATP conditions 16
S14 High PEG concentration promotes the reverse reaction 17

List of Tables

S1 RNA sequences used in this research 18
S2 Differences in each mode when M-RNA of length n (≥ 6) is cleaved 19

1 Comparison of degradation product distributions generated by RNase A and the Rnl1 reverse reaction

To determine whether the RNA degradation observed in the Rnl1 reaction products was caused by RNase contamination or by the reverse reaction of Rnl1, degradation products generated by RNase A were prepared and compared with the Rnl1 reaction products (Figure S1). Ribonuclease (DNase-free) Solution (Nippon Gene) was diluted 10-fold to final concentrations ranging from 1 to 0.0001 µg/mL in a total reaction volume of 10 µL. FAM-U20 and FAM-C20 were used as substrates at a final concentration of 3.3 µM, and samples were incubated for 30 min. Because complete termination of RNase A activity is technically difficult, 1 µL of 50 mM DTT was added to reduce enzymatic activity, and the samples were immediately subjected to electrophoresis. Rnl1 reaction products were prepared from FAM-U20 and FAM-C20 under the standard ligation conditions described in the Experimental section of the main text, except that the incubation time was 4 hours. The electrophoretic patterns of the RNase A and Rnl1 reaction products were then compared.

2 Evaluation of ligation and degradation efficiencies using ddC-modified RNA donors

To determine whether donor circularization occurred under the 1 mM ATP condition, donor RNAs with a $2',3'$-dideoxy modification were used (Figure S2). The buffer composition and all other reaction conditions were identical to those described for the evaluation of the reverse reaction of Rnl1 in the Experimental section of the main text.

3 Quantification of the residual 20 nt polyU, polyC, and polyA substrates

The decrease in the intensity of the 20 nt substrate band was quantified from gel images for FAM-U20, FAM-C20, and FAM-A20, in which RNA degradation by the reverse reaction of Rnl1 was observed (Figure S3). Three independent experiments ($n = 3$), including independent sample preparation and gel electrophoresis, were performed. Band regions were manually selected from the gel images using ImageJ, and the area and mean fluorescence intensity of each band were measured. The mean background fluorescence intensity was also determined. The total FAM fluorescence for each band was calculated by subtracting the mean background intensity from the mean band intensity and multiplying the resulting value by the band area. For each sample, time points were collected at 0 and 20 min under the 20 µM AMP condition. All other reaction conditions, including the buffer composition, were identical to those described in the Experimental section of the main text.

4 Estimation of the minimum degradation product length using 5 nt FAM-labeled polyU

To estimate the minimum size of the degradation products generated by Rnl1, FAM-U5, a 5 nt FAM-labeled polyU oligonucleotide, was used as a size reference and electrophoresed alongside FAM-U20 reacted under the standard ligation conditions described in the experimental section of the main text (Figure S4).

5 Evaluation of G residue degradation using DNA-RNA chimeric substrates

To determine whether the reduced reverse reaction observed for polyG was attributable to G-quadruplex formation, reverse reactions were examined using substrates designed to minimize G-quadruplex formation (Figure S5). Three oligonucleotides were used: FAM-dT17, a 17 nt deoxythymidine homopolymer; FAM-dT7-U3-dT7; and FAM-dT7-G3-dT7, in which three consecutive U or G residues were flanked by seven deoxythymidine residues on each side. The AMP concentration was varied among 0, 2, 20, and 200 µM. The buffer was prepared by mixing equal volumes of 10× basal buffer without ATP (500 mM Tris-HCl, pH 7.5, 100 mM $MgCl_2$, and 100 mM DTT) and AMP solution (Sigma-Aldrich), and was diluted to 1× in the reaction mixture. All other reaction conditions were identical to those described in the Experimental section of the main text.

6 Quantification of the staining efficiency of SYBR Green II for RNA homopolymers using a Qubit assay

To evaluate the staining bias and efficiency of SYBR Green II (Invitrogen) toward homopolymers, a Qubit-based fluorescence assay was performed (Figure S6). Six RNA samples were prepared: four single-stranded homopolymers (U20, C20, A20, and G20) and two equimolar mixtures of complementary strands (U20+A20 and C20+G20). For each sample, 100 ng of RNA was mixed with 199 µL of TBE-urea buffer containing SYBR Green II diluted 1:10,000. The final urea concentration was varied among 0, 0.5, 1, 2, 4, and 6 M. After incubation for 2 min at room temperature, fluorescence was measured using a Qubit 4 fluorometer (Invitrogen) with the Blue (470 nm) mode. Background fluorescence from the corresponding TBE-urea buffer without RNA was subtracted from each measurement, and the corrected fluorescence intensity was plotted against urea concentration.

7 Post-staining to detect products lacking the FAM label

For the experiment shown in Figure 2, post-staining with SYBR Green II was performed to detect products lacking the FAM label (Figure S7). DynaMarker Small RNA II Ladder Marker (BioDynamics Laboratory Inc.) was used as the RNA size marker. After electrophoresis, the gel was removed from the glass plates and immersed in 50 mL of 1× TBE buffer containing 3 µL of SYBR Green II. The gel was then post-stained for 30 min on an orbital shaker.

8 Unexpected RNA degradation in the absence of donor RNA under excess ATP conditions

As a control experiment, FAM-labeled RNA homopolymers (FAM-U20, FAM-C20, FAM-A20, FAM-G20) were incubated without donor RNA (Figure S8). ATP (magnesium salt, Sigma-Aldrich) concentration was tested at 1, 5, and 10 mM. ATP concentrations of 5 mM and 10 mM were prepared by adding 4 mM or 9 mM ATP (magnesium salt, Sigma-Aldrich) to T4 RNA Ligase buffer (TAKARA), which contains 1 mM ATP. All other reaction conditions were the same as described in the Experimental section of the main text.

9 Verification of the reverse reaction under different buffer conditions with or without ATP, AMP, and Mg^{2+}

The efficiency of the reverse reaction for FAM-U20 was examined in the presence or absence of ATP (Sigma-Aldrich), AMP (Sigma-Aldrich), and $MgCl_2$ (FUJIFILM Wako) (Figure S9). ATP and AMP were tested at concentrations of 1 mM and 2 µM, respectively, and $MgCl_2$ at 10 mM. The reactions were carried out in a 10× custom buffer containing 500 mM Tris-HCl and 100 mM DTT for 4 hours. All other reaction conditions were the same as described in the Experimental section of the main text.

10 Effects of low ATP concentrations on ligation and degradation by Rnl1

To investigate the effects of low ATP concentrations on both ligation and the reverse reaction, ATP concentrations of 0, 2, 20, and 200 µM were examined (Figure S10). Four substrate RNAs (FAM-U20, FAM-C20, FAM-A20, and FAM-G20) were reacted with their corresponding donor RNAs (pU19-ddC, pC19-ddC, pA19-ddC, and pG19-ddC, respectively). The donor RNAs were synthesized with a 2′,3′-dideoxy terminus to prevent donor circularization during the reaction. All other reaction conditions were identical to the Excess AMP conditions described in the Experimental section of the main text.

11 Temperature dependence of the Rnl1 reverse reaction under pH-stable conditions using MOPS buffer

To minimize temperature-dependent changes in pH, the reverse reaction was evaluated using MOPS buffer (Figure S11). MOPS buffer was adjusted to pH 7.5 with 2 M NaOH. A Tris-free 5× buffer containing 1 mM AMP, 50 mM $MgCl_2$, and 50 mM DTT was prepared and diluted to a 1× working concentration in the reaction mixture, resulting in final concentrations of 200 µM AMP, 10 mM $MgCl_2$, and 10 mM DTT. MOPS was added to a final concentration of 50 mM. All other reaction conditions were identical to those described in the Experimental section of the main text.

12 Stochastic simulation based on the Gillespie algorithm

In order to elucidate the reverse reaction mechanism of Rnl1 and distinguish between the nonprocessive exo mode [1,2], processive exo mode [3], and endo mode [4], we performed a stochastic simulation based on the Gillespie algorithm [5,6] (Figures 7c-d and S4). This computational model serves to complement experimental gel electrophoresis data (Figure 7a) by simulating the alteration in RNA polymer length over time in response to varying ligation and degradation conditions.

12.1 Simulation model for RNA ligation and degradation

The model is designed to track RNA species categorized according to their modification of 5′-end: FAM (6-carboxyfluorescein)-labeled RNA (FAM-RNA) and adenylated RNA (App-RNA). These modifications defined ligation capability; both FAM-RNA and App-RNA were designated as ligation acceptors (reacting via the hydroxyl group at 3′-end), yet only App-RNA was modeled as a donor capable of ligation. Moreover, the model posits that degradation is an AMP-dependent reverse reaction (Figure 4). Furthermore, it is hypothesized that the chemical reactions in this model adhere to irreversible elementary reaction kinetics as opposed to Michaelis-Menten kinetics. This approach enables the explicit tracking of AMP consumption and generation, thereby capturing the dynamic interplay between ligation and degradation processes observed in the monodisperse 20 nt RNA experiments. In this simulation, three degradation modes were defined for the degradation mechanism: nonprocessive exo

mode[1,2], processive exo mode[3], and endo mode[4]. The three modes are distinguished by the length and the number of App-RNA molecules produced by degradation (see Table S2). Our experiments yielded an observed minimum length of 4 nt for FAM-RNA, suggesting that the minimum length of the acceptor RNA produced by degradation was set to 4 nt across all modes. The endo mode generates a single App-RNA of any permissible length (l). When the length of the degraded RNA is n, l is drawn from a uniform distribution within the range $2 \leq l \leq n-4$.

Conversely, earlier studies have demonstrated that when the $3'$ end is a hydroxyl group, the transfer of at least 2 nt or 3 nt is substantiated, while the transfer of 1 nt remains unconfirmed[7]. Consequently, the length of the App-RNA produced by the nonprocessive exo mode and the processive exo mode was assumed to be 2 nt or 3 nt with equal probability. In the absence of quantitative evidence favoring either length, the two permissible lengths were assigned equal probability. However, when the reactant RNA length is 6 nt, the resulting adenylated RNA is restricted to 2 nt to satisfy the minimum acceptor length constraint.

A uniform distribution over all permissible App-RNA lengths was not introduced for the nonprocessive exo mode because it would make this mode indistinguishable from the endo mode under the present model representation: both would generate a single App-RNA with l drawn uniformly from $2 \leq l \leq n-4$ and an acceptor RNA of length $n-l$. This model-level equivalence does not imply that terminal and internal cleavage are biochemically identical; rather, the present model does not encode the cleavage position independently of product length.

The difference between the two exo modes is the number of product App-RNAs: the nonprocessive exo mode produces a single App-RNA per event, whereas the processive exo mode can generate multiple App-RNAs. In the processive exo mode, the continuation of cleavage is determined by Bernoulli trials with a success probability of $p = 0.5$.

12.2 Stochastic simulation using Gillespie algorithm

The system's time evolution was simulated using the Gillespie algorithm. The state of the system is defined by the vector of molecular counts for all RNA species and AMP. In each simulation step, the total reaction propensity $a_0 = \sum a_\mu$ is calculated based on the current population of species and the stochastic rate constants defined in the reaction scheme. To calculate the propensity of each reaction, the set of reactions in this model is defined by the following reaction equations:

$$\begin{aligned} \text{ligation}: \quad & M\text{-RNA}_n + \text{App-RNA}_m \xrightarrow{k_{\text{lig}}} M\text{-RNA}_{n+m} + \text{AMP} \ , \\ \text{degradation}: \quad & M\text{-RNA}_n + \text{AMP} \xrightarrow{k_{\text{deg}}} M\text{-RNA}_{n-l} + \text{App-RNA}_l \ , \end{aligned}$$

where, $M \in \{\text{FAM}, \text{App}\}$ represents the modification of $5'$ end, subscripts n, m, and l are natural numbers that denote the length of the RNA, and k_{lig} and k_{deg} are the rate constants or ligation and degradation, respectively. Then, the propensity function for ligation (a_{lig}) and degradation (a_{deg}) is calculated using the following formulas:

$$\begin{aligned} a_{\text{lig}} &= c_{\text{lig}} N_{M\text{-RNA}_n} N_{\text{App-RNA}_m} \ , & c_{\text{lig}} &= \frac{k_{\text{lig}}}{V} \ , \\ a_{\text{deg}} &= c_{\text{deg}} N_{M\text{-RNA}_n} N_{\text{AMP}} \ , & c_{\text{deg}} &= \frac{k_{\text{deg}}}{V} \ , \end{aligned}$$

where, c_{lig} and c_{deg} are stochastic rate constants of ligation and degradation, respectively, and V is the simulation volume.

Two random numbers (r_1, r_2) are generated to determine the time interval to the next reaction, $\tau = (1/a_0)\ln(1/r_1)$, and to select the specific reaction channel μ^* that satisfies inequality $\sum_{\nu=1}^{\mu^*-1} a_\nu < r_2 a_0 \leq \sum_{\nu=1}^{\mu^*} a_\nu$. The molecular counts are then updated according to the stoichiometry of the selected reaction, ensuring mass conservation.

12.3 Simulation setup and computational parameters

In this simulation, all variables and parameters are dimensionless. Furthermore, the simulations were initialized to replicate the molecular environment of the corresponding *in vitro* experiments. The initial molecular counts for AMP (N_{AMP}) and FAM-RNA$_{20}$ ($N_{\mathrm{FAM\text{-}RNA}_{20}}$) were set in 20×10^3 and 3.3×10^3 respectively. This ratio was determined based on experimental molar concentrations of 20 µM AMP and 3.3 µM FAM-RNA$_{20}$ to ensure a physically consistent representation of molecular density within the stochastic framework. At $t = 0$, the counts for all other RNA species were set to zero.

To focus on qualitative evaluation of the reaction mechanisms, the rate constants (k) for all reaction channels were set to unity ($k = 1$), and the simulation volume (V) was set to $V = 1 \times 10^{-6}$. In other words, all stochastic rate constants (c) were fixed at $c = 1 \times 10^{-6}$.

All simulations were executed over a time-span until a maximum simulation time (t_{max}) of 50 was reached. The molecular counts for all species were recorded at constant time intervals of $\Delta t = 0.01$. To ensure statistical significance and compensate for intrinsic noise, we executed an ensemble of 1000 independent trajectories for each condition. The results presented in Figure 7c-d and Figure S12a-c represent the ensemble averages of these trajectories.

13 Verification of the Rnl1 reverse reaction using miRNA substrates

To investigate whether the reverse reaction also occurs in natural RNA sequences rather than only in homopolymers, four representative miRNAs were examined (Figure S13). miR-21, a well-known oncogenic miRNA, miR-1, which has a low GC content, miR-124, which has a high GC content, and miR-145, which contains consecutive U and C residues, were selected as substrates and synthesized by FASMAC. Each miRNA was incubated for 1 hour in the presence of the donor substrate pA20 under either a control condition containing 200 µM AMP or the standard ligation condition containing 1 mM ATP. The 1 mM ATP condition was identical to the standard ligation conditions described in the Experimental section of the main text. For the 200 µM AMP condition, the AMP concentration was adjusted using a 5× AMP buffer. This buffer was prepared by mixing an equal volume of 10× basal buffer without ATP (500 mM Tris-HCl, pH 7.5, 100 mM $MgCl_2$, and 100 mM DTT) with an AMP solution (Sigma-Aldrich) and was diluted to 1× in the reaction mixture to yield a final AMP concentration of 200 µM. All other reaction conditions were identical to those described in the Experimental section of the main text.

14 Evaluation of the reverse reaction efficiency as a function of PEG concentration

To evaluate how PEG concentration influences the efficiency of the reverse reaction of FAM-U20, reactions were performed in the presence of PEG 8000 at concentrations from 0% to 25% (in 5% increments) (Figure S14). The AMP concentration was fixed at 200 µM using a 5× AMP buffer. This buffer was prepared by mixing the 10× basal buffer without ATP (500 mM Tris-HCl pH 7.5, 100 mM $MgCl_2$, and 100 mM DTT) with an equal volume of AMP solution (Sigma-Aldrich) and was diluted to 1× working concentration in the reaction. All other reaction conditions were the same as described in the Experimental section of the main text.

Supplementary figures

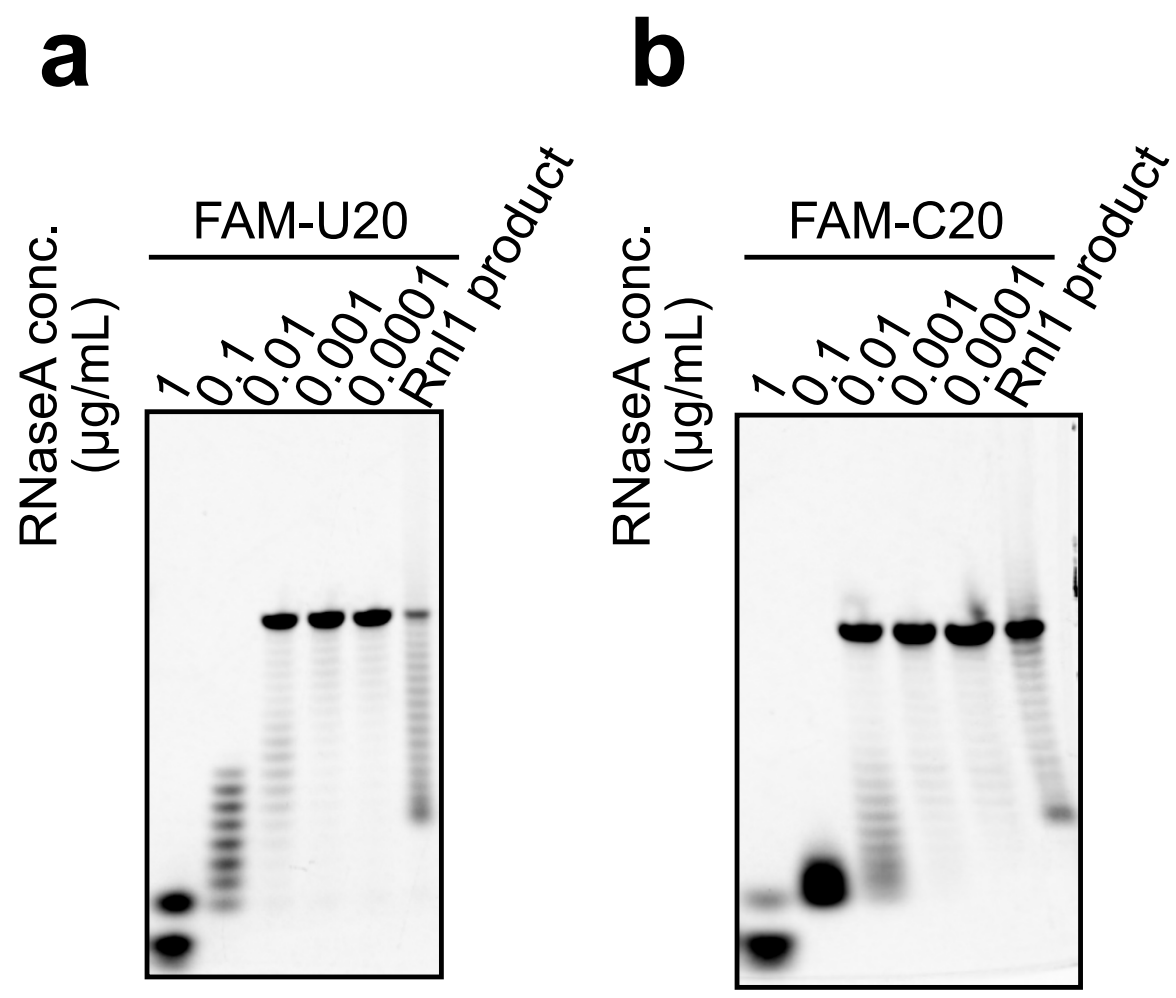

Figure S1. Comparison of the distribution of reaction products generated by RNase A and Rnl1 The degradation products generated by RNase A were examined over a concentration range of 1 to 0.0001 µg/mL. For comparison, FAM-U20 and FAM-C20 were incubated with Rnl1 under standard ligation conditions at 16°C for 4 h. The distribution of degradation products generated by RNase A differed markedly from that produced by the Rnl1 reverse reaction. These results suggest that the RNA degradation observed in the Rnl1 reaction is unlikely to be caused by RNase A contamination. Furthermore, the shortest degradation products generated by RNase A migrated below those generated by Rnl1, indicating that degradation by the Rnl1 reverse reaction does not proceed to complete degradation but instead terminates at intermediate-length products.

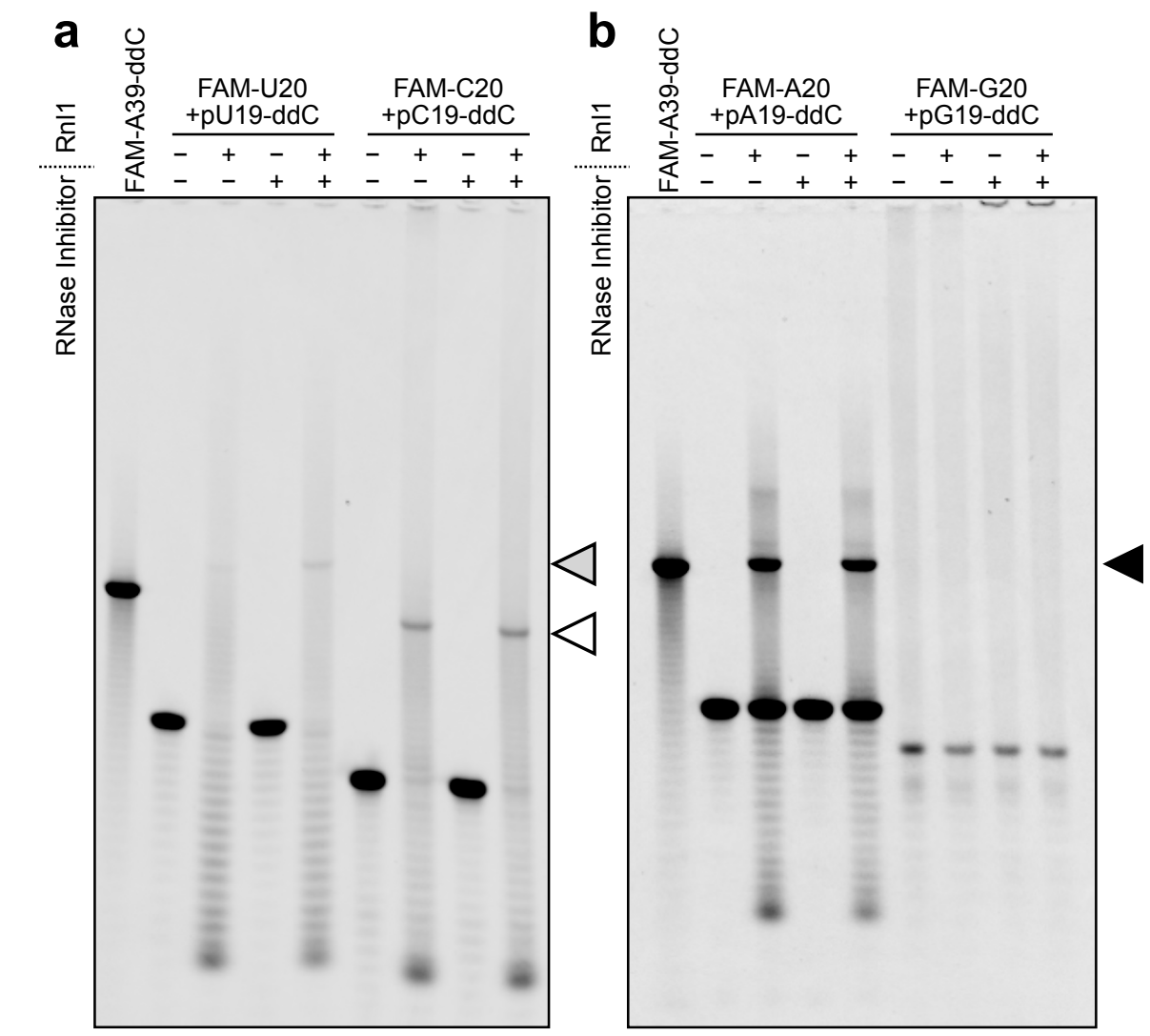

Figure S2. Analysis of the reverse reaction under circularization-deficient conditions using a ddC-modified donor
The reactions were performed at 16°C for 1 h under 1 mM ATP conditions. Intermolecular ligation products were detected for polyU and polyC when circularization was prevented by using a 3′-terminal ddC-modified donor. Their migration position was consistent with that of the FAM-A39-ddC size marker, confirming that the observed ligation products were approximately 40 nt in length. Under the original reaction conditions (Figure 2), no corresponding intermolecular ligation products were detected, suggesting that intramolecular circularization is favored over intermolecular ligation. Arrows indicate bands corresponding to approximately 40 nt ligation products: gray arrow indicates polyU, white arrow indicates polyC, and black arrow indicates polyA.

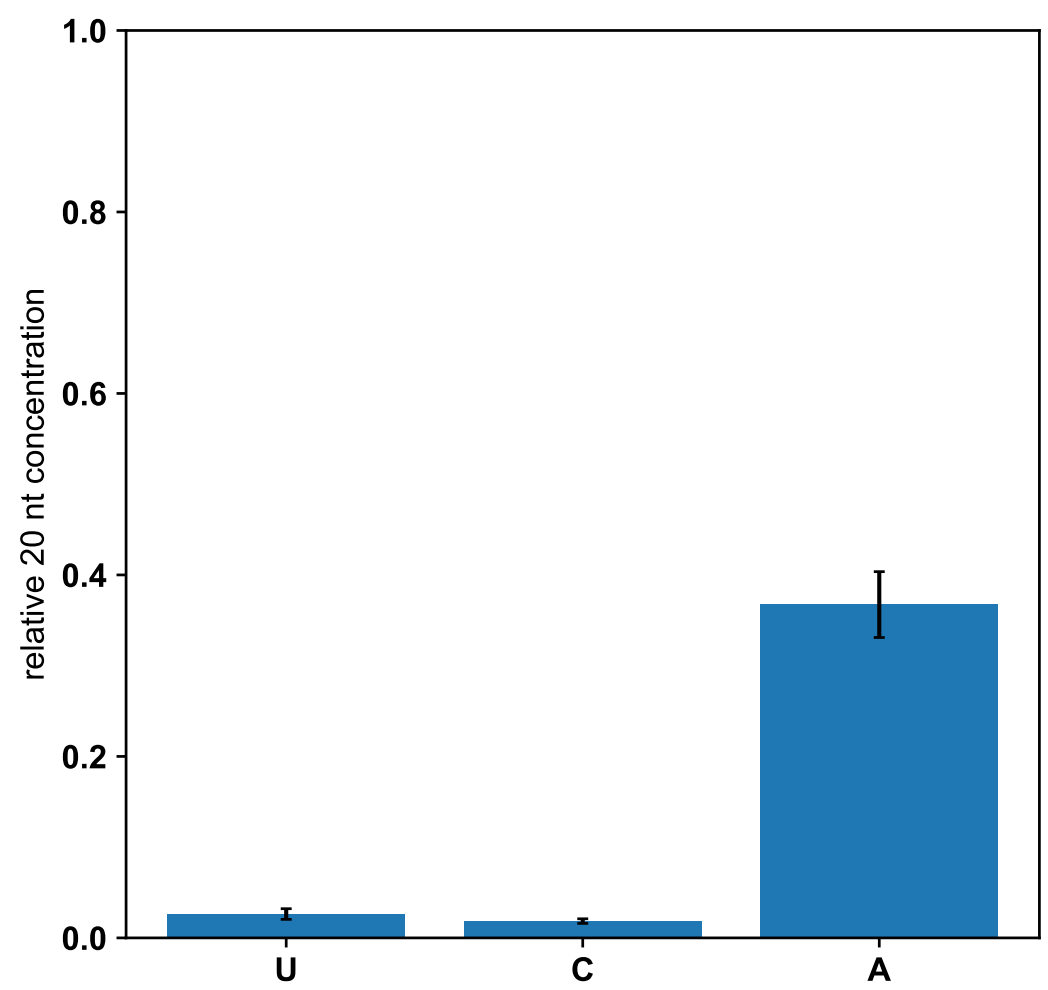

Figure S3. Analysis of substrate reduction under donor-absent 20 µM AMP conditions
Under donor-absent 20 µM AMP conditions, the reduction of the substrate band after 20 min of reaction was quantified from gel images. The difference in substrate reduction between polyU and polyC was less pronounced than under ATP conditions.

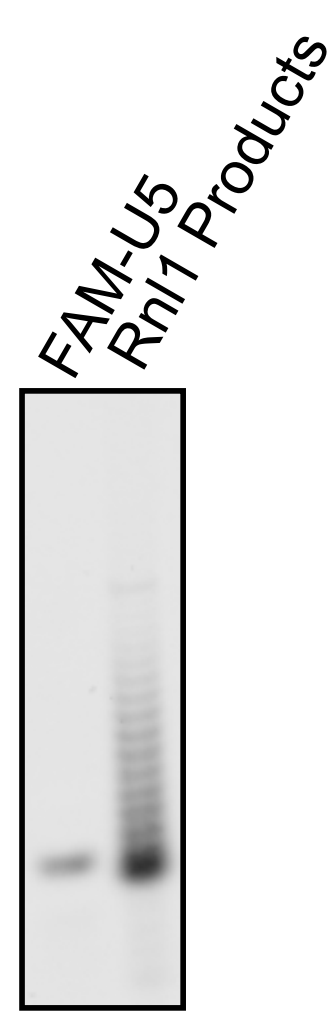

Figure S4. Comparison of the migration of FAM-U5 and Rnl1 reaction products
The shortest Rnl1 reaction products migrated at approximately the same position as FAM-U5, indicating that degradation by the Rnl1 reverse reaction terminates at approximately 4–5 nt. Reactions were performed with 1 mM ATP at 16°C for 1 h.

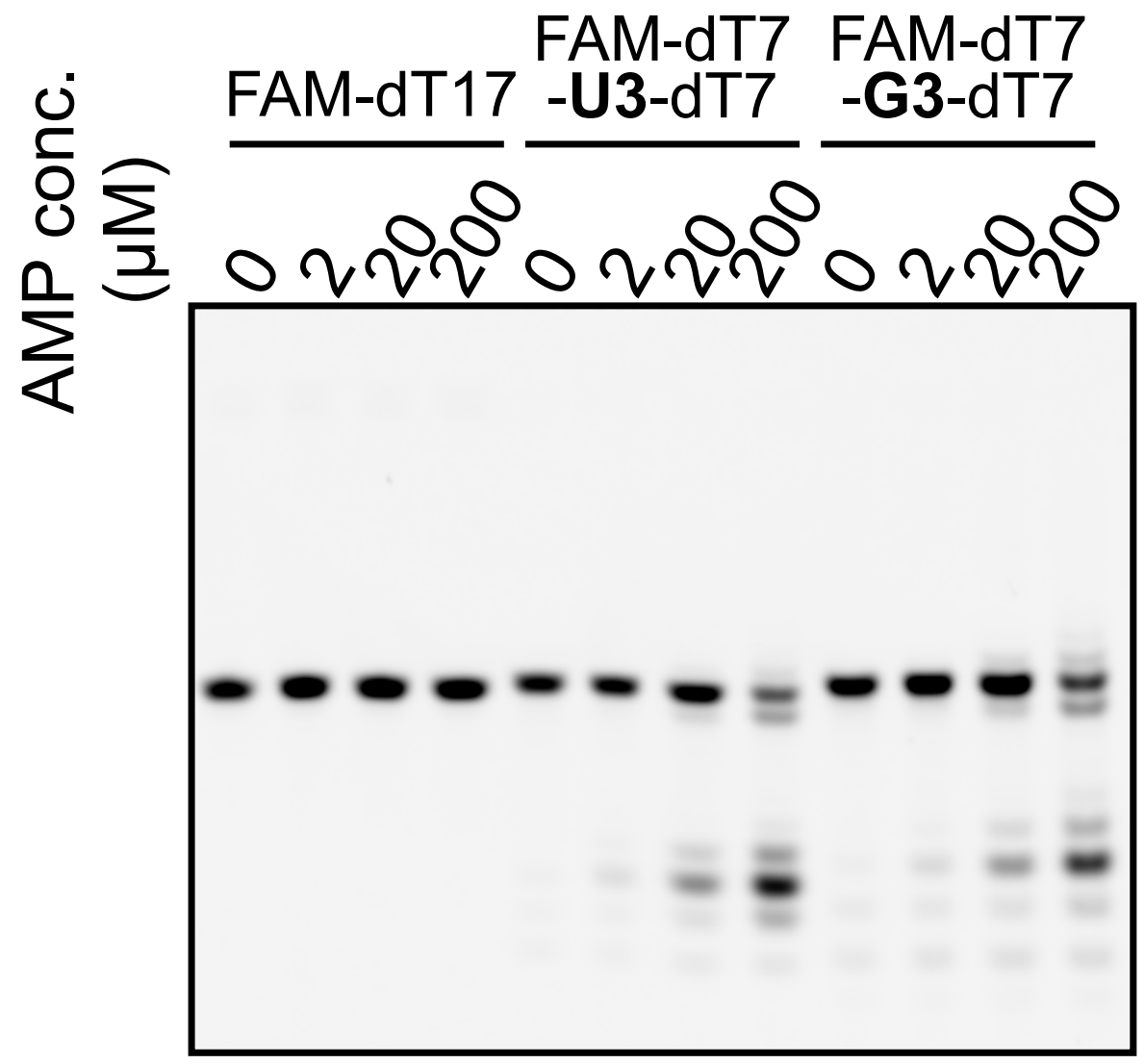

Figure S5. Degradation of guanine-containing chimeric DNA-RNA substrates
Reactions were performed at 16°C for 1 h, with AMP concentrations of 0, 2, 20, and 200 µM. FAM-dT17 showed no detectable degradation, even in the presence of 200 µM AMP. In contrast, degradation was observed for chimeric substrates containing three consecutive U or G residues flanked by dT residues. These results suggest that guanine-containing substrates can undergo degradation when the sequence is less prone to forming secondary structures such as G-quadruplexes. Furthermore, the observation of degradation products from the chimeric substrates suggests that the Rnl1 reverse reaction may proceed through an endonuclease-like cleavage mechanism, in which cleavage occurs within the RNA strand rather than exclusively from the termini.

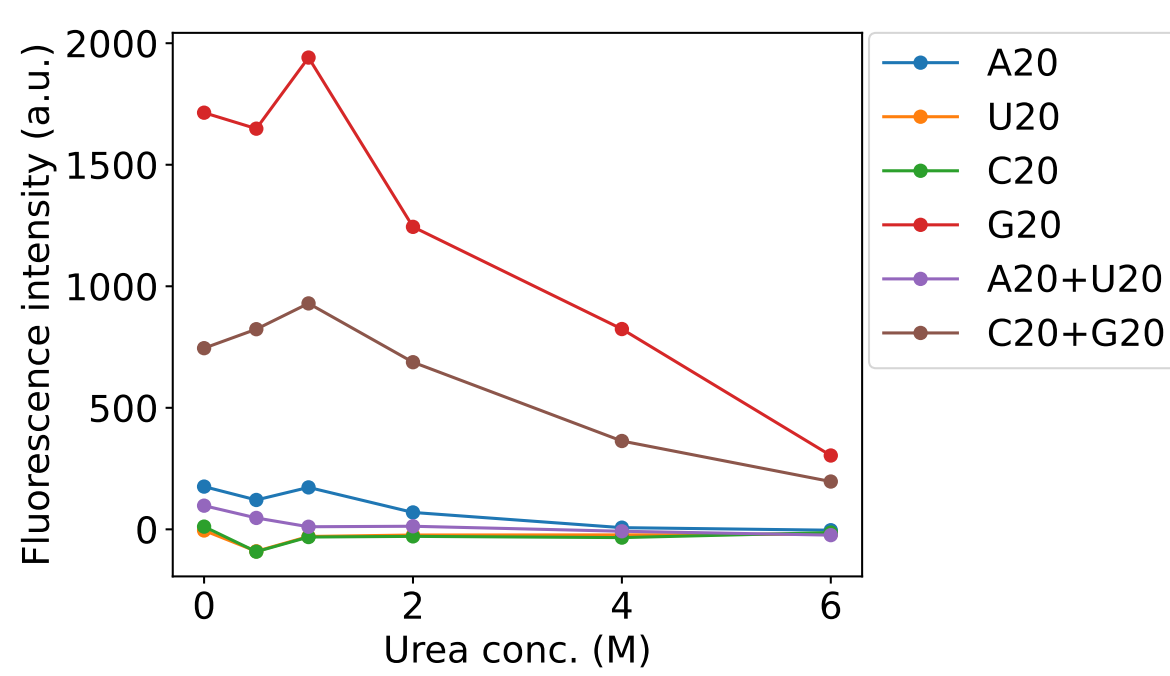

Figure S6. Evaluation of the sequence-dependent fluorescence bias of SYBR Green II using a Qubit fluorometer
For the fluorescence assay, 100 ng of RNA was mixed with TBE-urea buffer containing SYBR Green II diluted 1:10,000, with the final urea concentrations of 0, 0.5, 1, 2, 4, and 6 M. Samples were incubated for 2 min at room temperature before fluorescence measurement using a Qubit 4 fluorometer. SYBR Green II exhibited markedly higher fluorescence for polyG than for the other homopolymers, whereas the fluorescence responses for polyU and polyC were substantially lower. These results demonstrate a strong sequence-dependent bias in the fluorescence response of SYBR Green II.

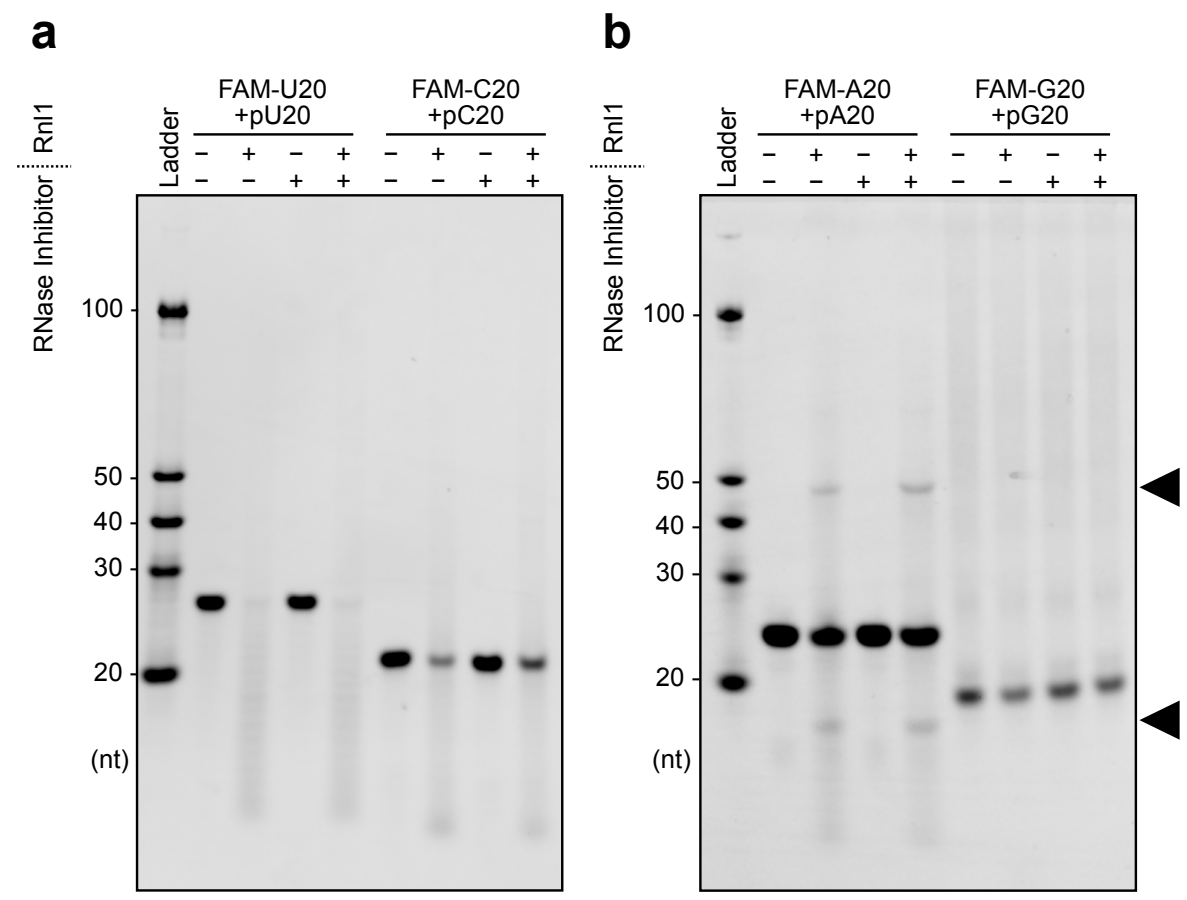

Figure S7. SYBR Green II post-staining of the gel shown in Figure 2
The gel shown in Figure 2 was post-stained with SYBR Green II to visualize unlabeled reaction products. No additional bands corresponding to putative circularization products were observed for polyU or polyC. In contrast, an additional band migrating below the 20 nt substrate was observed for polyA, which may represent a circularization product. The black arrows indicate the products observed for polyA: the upper arrow indicates the approximately 40 nt ligation product, and the lower arrow indicates the putative circularization product.

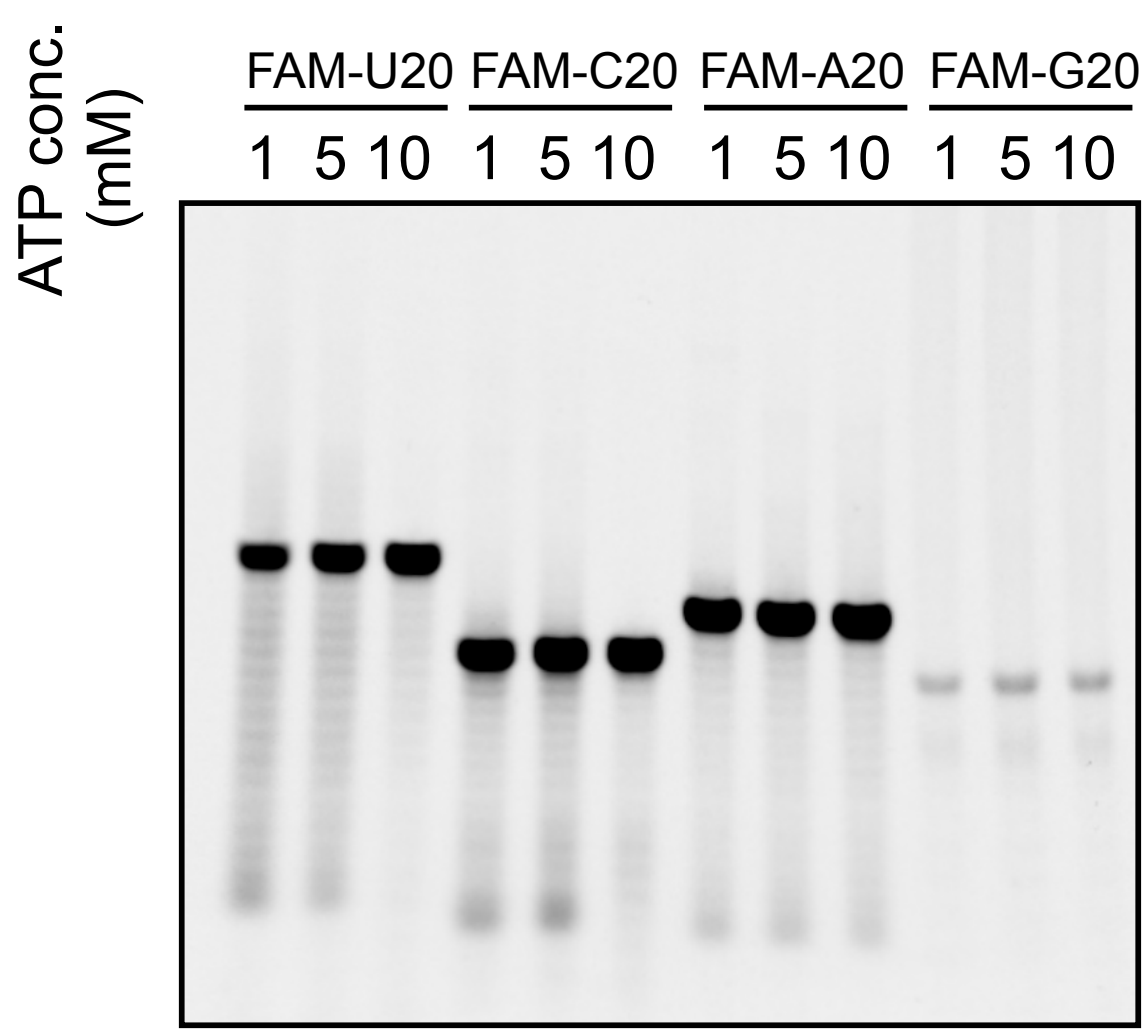

Figure S8. Control assays evaluating the effects of ATP concentration in the absence of a donor substrate
ATP concentrations of 1, 5, and 10 mM were tested at 16°C for 1 h. Even in the absence of donor RNA, minimal degradation signals were detected under excess ATP conditions. This observation is unexpected because degradation requires AMP [7], which is normally produced as a byproduct of ligation. In donor-absent reactions, ligation should not occur, and thus AMP should theoretically be absent. A possible source of AMP in this experiment is its accidental release from adenylated Rnl1 (Figure 1(i)). Additionally, although not directly detectable, delabeled RNA may have been present in the reaction mixture. Once the FAM label is lost, such RNA could serve as a donor, generating AMP and enabling further degradation. Because AMP can be reused in the reverse reaction pathway, even small amounts could continuously drive additional degradation.

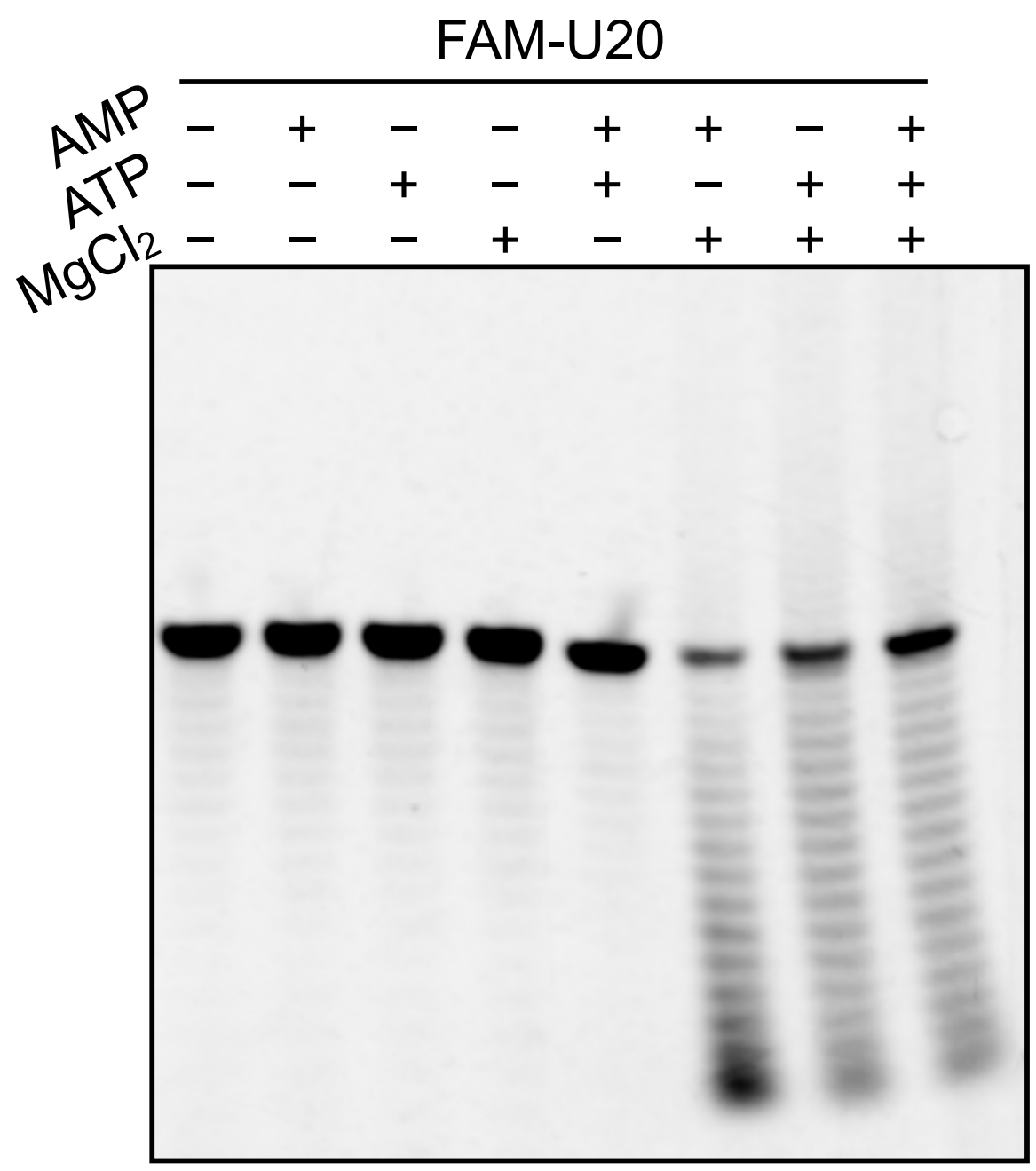

Figure S9. Effects of ATP, AMP, and Mg on the reverse reaction
The efficiency of the reverse reaction was examined in the presence or absence of ATP (1 mM), AMP (2 µM), and $MgCl_2$ (10 mM). The reactions were carried out at 16°C for 4 h. The results indicated that the reverse reaction did not occur in the presence of ATP alone, AMP alone, or magnesium alone. Furthermore, in the absence of magnesium, the reverse reaction was not observed under any tested conditions.

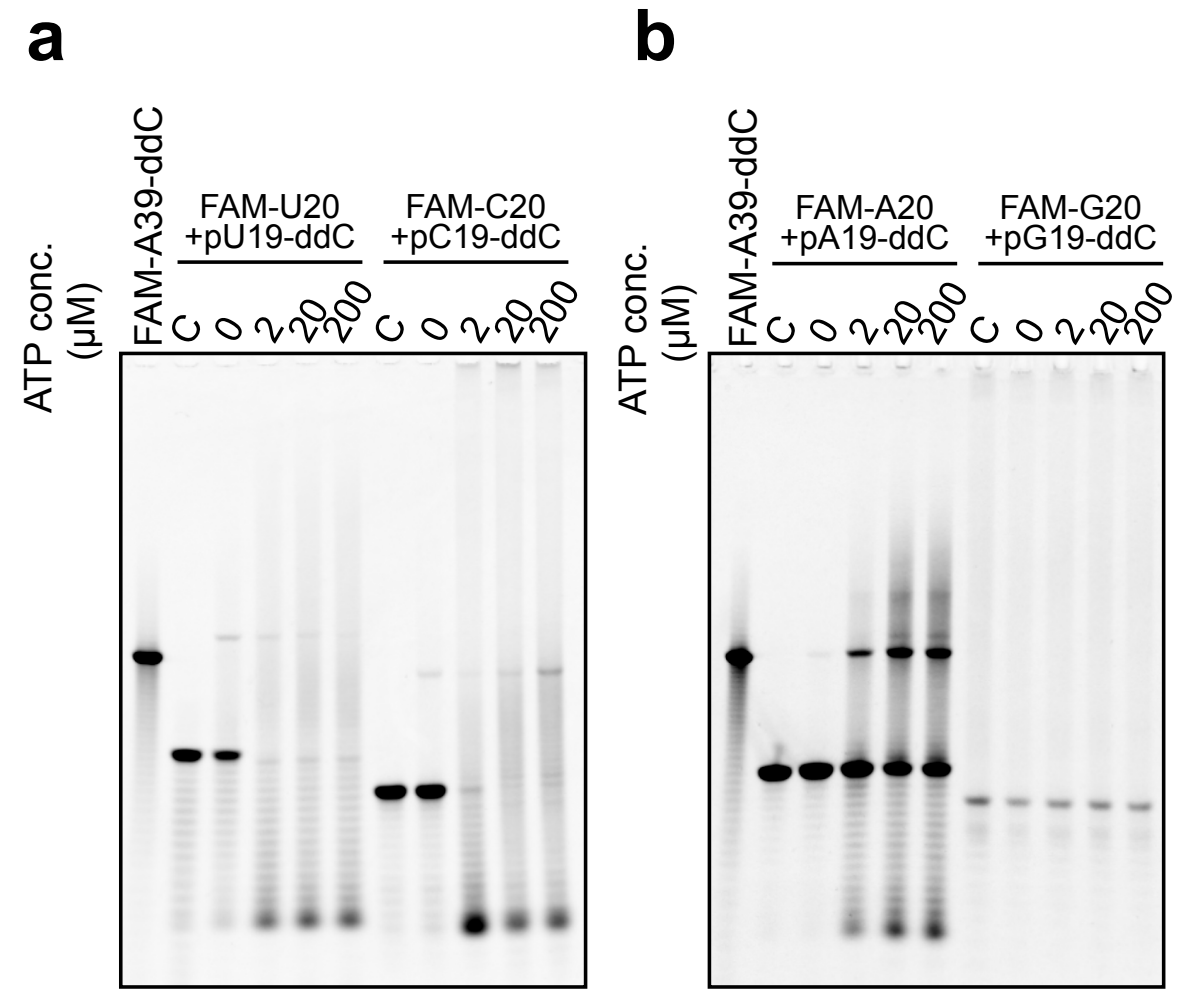

Figure S10. Effects of low ATP concentrations on ligation and degradation using a donor with a 3′-terminal ddC modification
Lane C represents the control reaction performed in the absence of both ATP and donor RNA. Ligation and degradation products were detected under ATP concentrations of 0, 2, 20, and 200 µM. In particular, polyA showed an increase in ligation products with increasing ATP concentration. Notably, both ligation and degradation products were also detected in the absence of ATP. These products may result from trace amounts of pre-adenylated Rnl1 present in the commercial enzyme preparation; however, the underlying mechanism remains to be clarified.

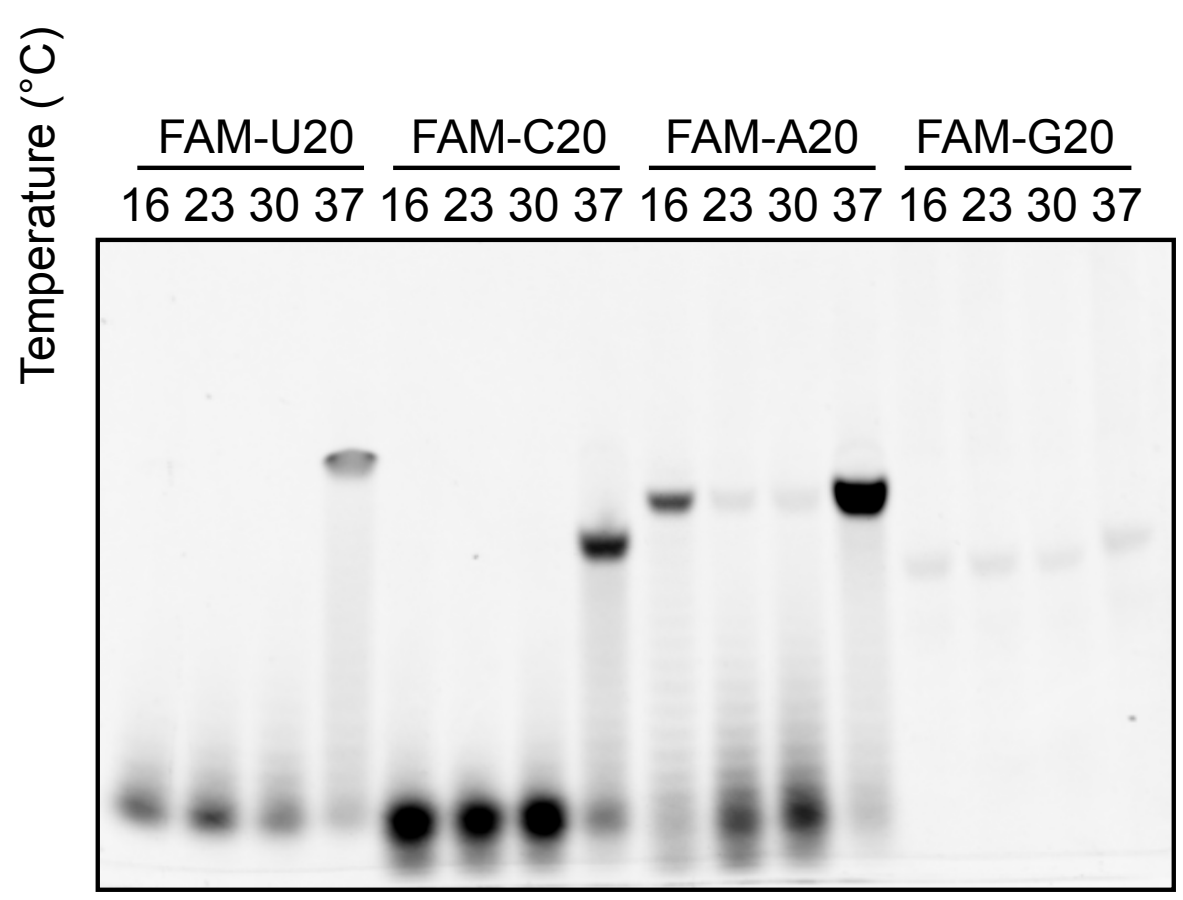

Figure S11. Temperature dependence of the Rnl1 reverse reaction in MOPS buffer
Temperature-dependent degradation was examined using MOPS buffer, which minimizes temperature-dependent pH changes. Reactions were performed at 16, 23, 30, and 37°C for 1 h. The overall trend was comparable to that observed in Tris buffer (Figure 5), suggesting that the suppression of degradation at 37°C is unlikely to be caused by pH changes and is instead primarily attributable to temperature.

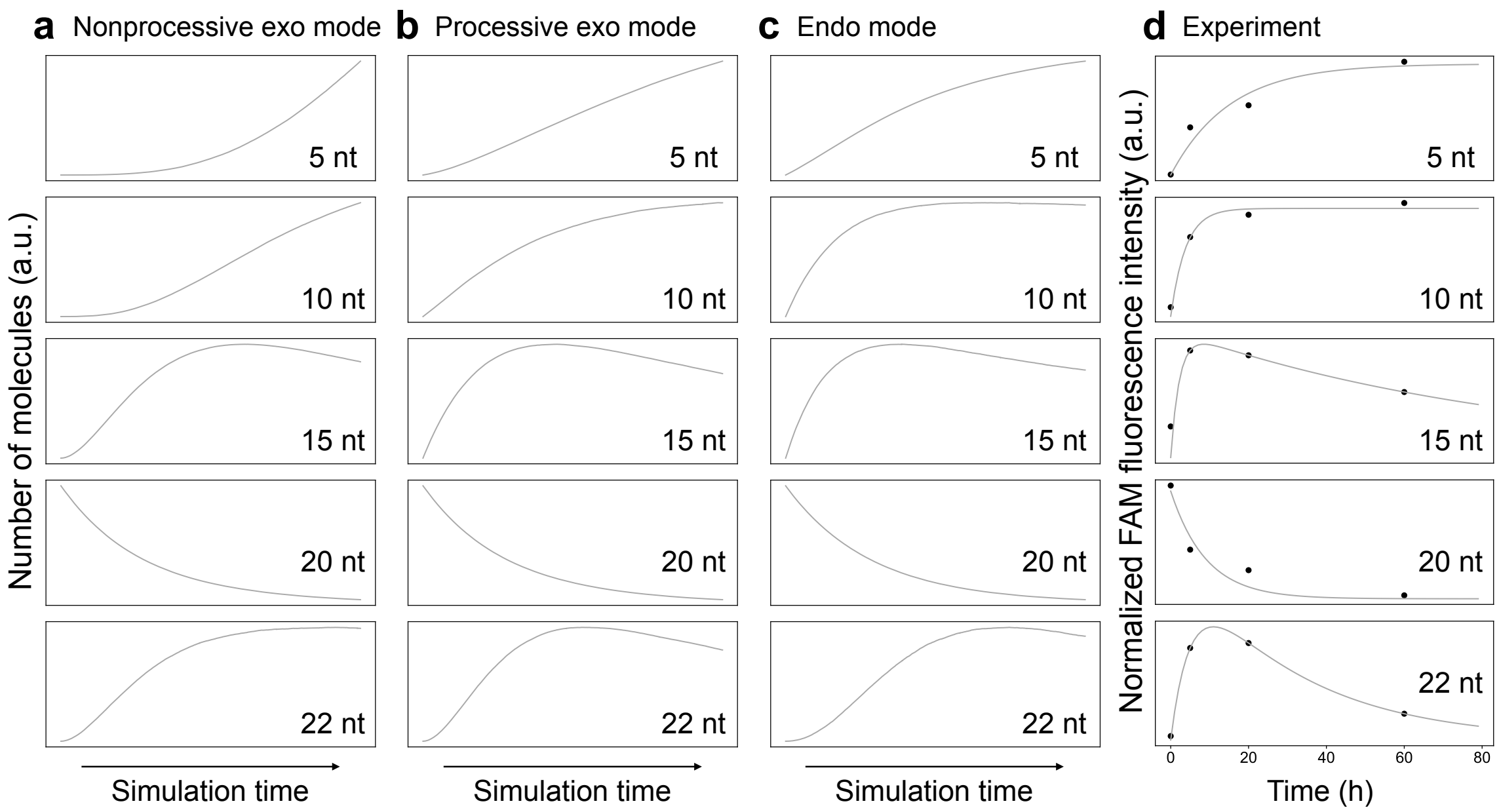

Figure S12. Stochastic simulations of Rnl1-mediated reverse-reaction modes and quantitative gel-based kinetics

(a–c) Stochastic simulations of RNA length distributions for three proposed reverse-reaction modes (Figure 7): (a) nonprocessive exo mode, (b) processive exo mode, and (c) endo mode. The bottom axis indicates the simulation time, and the left axis indicates the normalized number of molecules. (d) Quantitative analysis and function fitting of Rnl1-mediated FAM-U20 degradation based on gel electrophoresis from Figure 7a. From the gel images, the time-dependent band intensities corresponding to polymer species of 5, 10, 15, and 20 nt were analyzed as representative length classes, and the 22 nt species was additionally analyzed as a representative re-ligation product. The bottom indicates time (h), and the left indicates normalized FAM fluorescence intensity (a.u.). Band intensities were fitted to extract time-dependent changes in selected species.

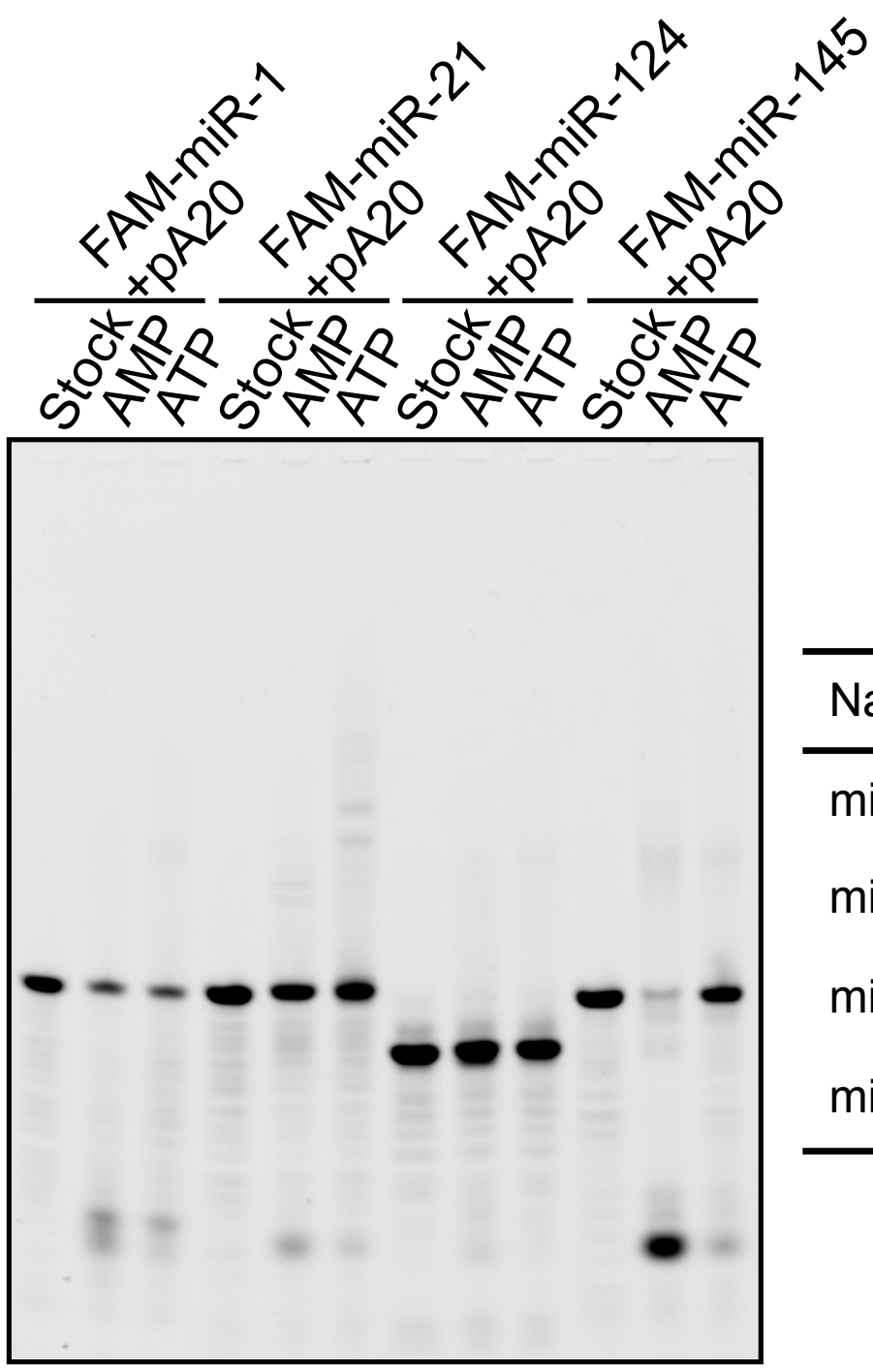

Name	Sequence	GC content
miR-1	UGGAAUGUAAAGAAGUAUGUAU	27.27%
miR-21	UAGCUUAUCAGACUGAUGUUGA	36.36%
miR-124	UAAGGCACGCGGUGAAUGCC	60.00%
miR-145	GUCCAGUUUUCCCAGGAAUCCCU	52.17%

Figure S13. Ligation and degradation of miRNAs under AMP and ATP conditions
Reactions were performed using FAM-labeled miRNAs in the presence of pA20 under 200 µM AMP or 1 mM ATP conditions at 16°C for 1 h. The sequence and GC content of miRNAs are summarized in the table. Degradation products were detected for FAM-miR-1, FAM-miR-21, and FAM-miR-145. The degradation products observed under 1 mM ATP migrated to positions similar to those observed under 200 µM AMP, supporting the interpretation that the degradation was caused by the Rnl1 reverse reaction. In contrast, no degradation products were detected for the GC-rich substrate FAM-miR-124.

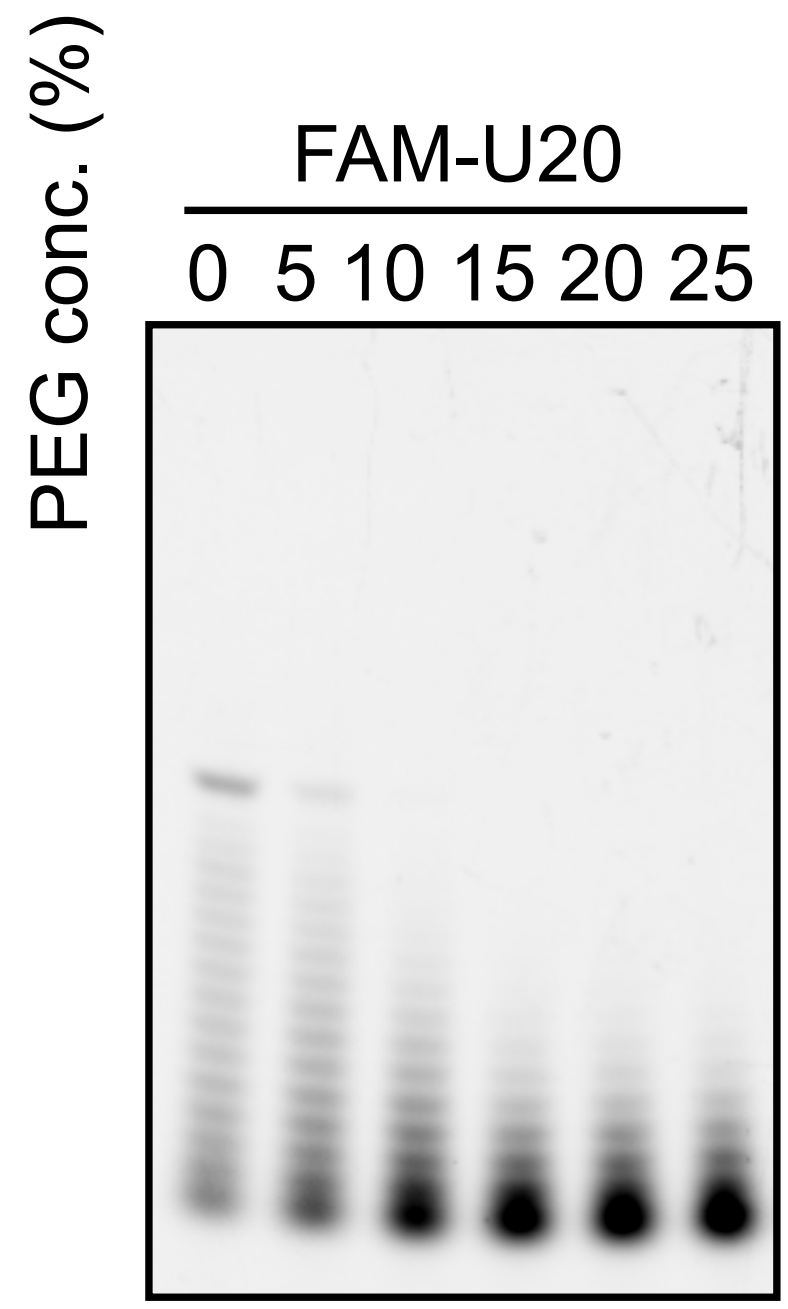

Figure S14. High PEG concentration promotes the reverse reaction
Reactions were performed with PEG concentrations ranging from 0% to 25% in 5% increments to examine how PEG affects the efficiency of the reverse reaction. Reactions were performed at 16°C for 1 h. The results showed that the reverse reaction proceeded more efficiently at higher PEG concentrations. However, even in the absence of PEG, the reverse reaction occurred. The occurrence of the reverse reaction without PEG suggests that PEG is not the direct cause of the reaction.

Supplementary tables

Table S1. RNA sequences used in this research

Name	Sequence ($5' \rightarrow 3'$)	Acceptor	Donor	Fluorescence (FAM-label)
FAM-U20	FAM-p-UUUUUUUUUUUUUUUUUUUU	◯		◯
FAM-C20	FAM-p-CCCCCCCCCCCCCCCCCCCC	◯		◯
FAM-A20	FAM-p-AAAAAAAAAAAAAAAAAAAA	◯		◯
FAM-G20	FAM-p-GGGGGGGGGGGGGGGGGGGG	◯		◯
pU20	p-UUUUUUUUUUUUUUUUUUUU	◯	◯	
pC20	p-CCCCCCCCCCCCCCCCCCCC	◯	◯	
pA20	p-AAAAAAAAAAAAAAAAAAAA	◯	◯	
pG20	p-GGGGGGGGGGGGGGGGGGGG	◯	◯	
U20	UUUUUUUUUUUUUUUUUUUU	◯		
A20	AAAAAAAAAAAAAAAAAAAA	◯		

Table S2. Differences in each mode when M-RNA of length n (≥ 6) is cleaved

	length (l) of App-RNAs produced	the number of App-RNAs produced
nonprocessive exo	$l = 2, 3$	1
processive exo	$l = 2, 3$	≥ 1 (Determined by Bernoulli trials)
endo	$2 \leq l \leq n - 4$ (Determined by uniform distribution)	1

References

[1] M. Bhagwat, L. J. Hobbs, N. G. Nossal, Journal of Biological Chemistry **1997**, 272, 28523.

[2] F. W. Perrino, S. Harvey, S. McMillin, T. Hollis, Journal of Biological Chemistry **2005**, 280, 15212.

[3] Z.-F. Cheng, M. P. Deutscher, Journal of Biological Chemistry **2002**, 277, 21624.

[4] T. K. Misra, S. Rhee, D. Apirion, Journal of Biological Chemistry **1976**, 251, 7669.

[5] D. T. Gillespie, Journal of Computational Physics **1976**, 22, 403.

[6] D. T. Gillespie, The Journal of Physical Chemistry **1977**, 81, 2340.

[7] M. Krug, O. C. Uhlenbeck, Biochemistry **1982**, 21, 1858.
